# Self-Reported Resilience During the COVID-19 Pandemic

**DOI:** 10.1001/jamanetworkopen.2025.20360

**Published:** 2025-07-16

**Authors:** Oluwabunmi Ogungbe, Tianyou Wang, Pallavi P. Balte, Sarah E. Slone, Diane Meyer, Norrina Bai Allen, Russell G. Buhr, Jana A. Hirsch, Karen Hinckley Stukovsky, Anna Kucharska-Newton, Kelley Pettee Gabriel, Elizabeth A. Regan, Vanessa Xanthakis, Carmen R. Isasi, Gregory Talavera, Martha Daviglus, Krista M. Perreira, Mario Sims, Jose Gutierrez Contreras, Namratha R. Kandula, Joyce S. Lee, Virginia J. Howard, Suzanne E. Judd, Prescott Woodruff, Victor E. Ortega, Amanda M. Fretts, Sally E. Wenzel, Wanda Phipatanakul, Nirupama Putcha, Nadia Hansel, Elizabeth Oelsner, Wendy S. Post

**Affiliations:** 1 Johns Hopkins School of Nursing, Baltimore, Maryland; 2 Johns Hopkins Bloomberg School of Public Health, Baltimore, Maryland; 3 Division of General Medicine, Department of Medicine, Columbia University Irving Medical Center, New York, New York; 4 Center for Epidemiology and Population Health, Northwestern Feinberg School of Medicine, Chicago, Illinois; 5 David Geffen School of Medicine, University of California, Los Angeles; 6 Fielding School of Public Health, University of California, Los Angeles; 7 Center for the Study of Healthcare Innovation, Implementation, and Policy, Greater Los Angeles Veterans Affairs Healthcare System, Los Angeles, California; 8 Dornsife School of Public Health, Drexel University, Philadelphia, Pennsylvania; 9 Department of Biostatistics, School of Public Health, University of Washington, Seattle; 10 Department of Epidemiology, University of North Carolina at Chapel Hill, Chapel Hill; 11 Department of Epidemiology, School of Public Health, University of Alabama at Birmingham, Birmingham; 12 Division of Rheumatology, Department of Medicine, National Jewish Health, Denver, Colorado; 13 Section of Preventive Medicine and Epidemiology, Department of Medicine, Boston University School of Medicine, Boston, Massachusetts; 14 Department of Biostatistics, Boston University School of Public Health, Boston, Massachusetts; 15 Department of Epidemiology and Population Health, Albert Einstein College of Medicine, Bronx, New York; 16 Graduate School of Public Health, San Diego State University, San Diego, California; 17 Institute for Minority Health Research, University of Illinois College of Medicine, Chicago; 18 Department of Social Medicine University of North Carolina School of Medicine, Chapel Hill; 19 Department of Social Medicine, Population and Public Health, School of Medicine, University of California, Riverside; 20 Department of Medicine, Northwestern Feinberg School of Medicine, Chicago, Illinois; 21 Division of Pulmonary Sciences and Critical Care Medicine, Department of Medicine, University of Colorado Anschutz Medical Campus, Aurora; 22 Department of Health Behavior, School of Public Health, University of Alabama at Birmingham, Birmingham; 23 Division of Pulmonary, Critical Care, Allergy and Sleep Medicine, Department of Medicine, University of California, San Francisco; 24 Division of Pulmonary Medicine, Department of Medicine, Mayo Clinic, Phoenix, Arizona; 25 Deptartment of Environmental and Occupational Health, School of Public Health, University of Pittsburgh, Pittsburgh, Pennsylvania; 26 Clinical Research Center, Boston Children’s Hospital, Boston, Massachusetts; 27 Harvard Medical School, Boston, Massachusetts; 28 Division of Cardiology, Department of Medicine, Johns Hopkins School of Medicine, Baltimore, Maryland

## Abstract

**Importance:**

Identifying factors associated with resilience during the COVID-19 pandemic can inform targeted interventions and resource allocation for groups disproportionately affected by systemic inequities.

**Objective:**

To examine factors associated with self-reported resilience during the COVID-19 pandemic in racially and ethnically diverse, community-dwelling US adults.

**Design, Setting, and Participants:**

This cross-sectional study was conducted as part of the Collaborative Cohort of Cohorts for COVID-19 Research (C4R) study, which assessed the associations of the pandemic with self-reported resilience of participants from 14 established US prospective cohorts since January 2021. This report includes participants who responded to the self-reported resilience question on C4R questionnaires. Data was initially analyzed from October 2023 to May 2024, with updated analyses performed from August 2024 to April 2025.

**Exposure:**

Race and ethnicity, behavior factors, health conditions, and social determinants of health measurements accessed before and during the COVID-19 pandemic through cohort visits and C4R questionnaires.

**Main Outcomes and Measures:**

Self-reported resilience was collected via 1 question (from the Brief Resilience Scale) in C4R questionnaires, “I tend to bounce back quickly after hard times.” Participants who answered agree or strongly agree were classified as resilient, and those who reported neutral, disagree, or strongly disagree were classified as not resilient. Modified Poisson regression was performed to estimate prevalence ratios (PRs) and access multivariable-adjusted associations with resilience.

**Results:**

Of 31 045 participants (18 672 [60%] women; 10 746 [34.6%] aged <65 years), 1185 (3.8%) identified as American Indian, 6728 (21.7%) as Black, 293 (0.9%) as East Asian, 6311 (20.3%) as Hispanic, 565 (1.8%) as South Asian, and 15 961 (51.3%) as White; a total of 23 103 participants (74.4%) self-identified as resilient. Compared with White participants, Black and Hispanic participants had higher prevalence of self-reported resilience (adjusted PR [aPR], 1.04; 95% CI, 1.02-1.06; aPR, 1.08; 95% CI, 1.06-1.11; respectively) and American Indian and East Asian participants had lower prevalence (aPR, 0.90; 95% CI, 0.86-0.94; aPR, 0.76; 95% CI, 0.68-0.84; respectively). Higher education, being married or living as married, higher income, and overweight were also associated with higher prevalence of resilience. Being female, having diabetes, and being unemployed were associated with lower prevalence of self-reported resilience. Compared with participants with public insurance only, participants with private insurance had higher prevalence of resilience (aPR, 1.07; 95% CI, 1.03-1.10). COVID-19 vaccination and infection statuses were not significantly associated with resilience. Modification analyses showed important racial and ethnic differences in how factors such as hypertension, marital status, and insurance status were associated with resilience.

**Conclusions and Relevance:**

In this cross-sectional study of 31 045 adults, self-reported resilience varied by race, ethnicity, and sociodemographic factors. These findings highlight the complex interplay of individual and social factors in shaping the perception of resilience.

## Key Points

**Question:**

What factors are associated with self-reported resilience during the COVID-19 pandemic across racial and ethnic groups in a US cohort?

**Findings:**

In this cross-sectional study of 31 045 participants from 14 US prospective cohorts, 74.4% of participants self-reported resilience. Black and Hispanic participants had statistically significantly higher prevalence ratios of resilience compared with White participants, while American Indian and East Asian participants had lower prevalence ratios (10% and 24%); higher education, being married, higher income, and private insurance were associated with greater resilience.

**Meaning:**

These findings suggest that resilience is shaped by individual and structural factors, and these findings can guide targeted interventions for future public health crises.

## Teaser

This cross-sectional study examines factors associated with self-reported resilience during the COVID-19 pandemic in racially and ethnically diverse, community-dwelling adults from 14 prospective US cohorts.

## Introduction

Resilience, defined as overcoming adversity or bouncing back from hardship, is linked to health and well-being.^1,2,3^ Acute and chronic stress can accelerate biological aging and chronic disease risk through inflammatory, metabolic, and behavioral pathways.^4,5^ Resilience capabilities that mitigate distress severity, support stress recovery, and promote wellness behaviors may influence such downstream effects.^6,7,8^ For example, higher resilience has been associated with reduced hypertension risk over 10 years in middle-aged adults.^9,10,11^ Psychosocial resilience may be a promising target for improving stress-related health outcomes.^12^

The COVID-19 pandemic came with prolonged, widespread hardship spanning health, economic, and social spheres.^13,14,15^ Whereas qualitative studies have explored individual experiences of resilience during the pandemic,^16^ quantitative indicators of coping capacity and resilience manifestations remain underexplored. The impact of the COVID-19 pandemic has been particularly severe among racial and ethnic groups disproportionately affected by systemic inequities, who faced greater burdens of chronic diseases, social strain, and limited public health resources. Consequently, these populations have experienced disproportionately higher rates of COVID-19 infection, hospitalization, and mortality.^17^ Disparities are rooted in longstanding systemic inequities, including unequal access to health care, higher rates of underlying health conditions, and socioeconomic disadvantages, such as employment in essential worker positions and crowded living conditions.^18,19^ Such disparities may influence resilience in complex ways: while the added burden of pandemic-related stressors and social strain could diminish resilience, histories of overcoming adversity and strong community ties might instead foster it.^20^ Understanding these dynamics is crucial for developing focused interventions, strengthening support systems, and guiding resource allocation during future disaster events.

Previous research on resilience has shown inconsistent patterns across demographic groups, with findings varying by measurement approaches and contextual factors. Some studies suggest that socioeconomically disadvantaged groups demonstrate greater resilience despite facing greater adversities,^21^ while others report lower resilience using different measurement tools.^22^ These mixed findings likely reflect both methodological differences and the complex interplay between individual capacities and structural resources. While previous studies on resilience have predominantly focused on clinical populations with chronic illness or mental health conditions, our study examines resilience in a large, population-based sample across multiple US regions, representing one of the most extensive racially and ethnically diverse assessments of resilience during the COVID-19 pandemic in the US. Resilience may also manifest differently across life stages and cultural contexts, highlighting the need to consider both individual and social determinants.^23^ Additionally, measuring resilience presents challenges due to its complex nature. Researchers have conceptualized resilience diversely—as a personality trait, a dynamic process, or a socioecological phenomenon—leading to varied assessment approaches.^24^ In this study, we specifically focus on the bounce-back dimension of resilience, ie, the self-perceived ability to recover from adversity, which represents the core element of resilience as conceptualized by the Brief Resilience Scale (BRS).

We used data from 14 US prospective cohorts participating in the Cohort of Cohorts for COVID-19 Research (C4R) to assess correlates of self-reported resilience during the COVID-19 pandemic. To inform future resource allocation and support during crisis, we examined differences in resilience and its associated factors across major racial and ethnic groups.

## Methods

### Study Sample

This cross-sectional study leveraged data from the C4R study, which includes 14 established US prospective cohort studies^25^ initially designed to study cardiovascular, pulmonary, and neurological health: Atherosclerosis Risk in Communities,^26,27^ Coronary Artery Risk Development in Young Adults,^28^ Genetic Epidemiology of COPD,^29^ Framingham Heart Study,^30^ Hispanic Community Health Study/Study of Latinos,^31,32^ Jackson Heart Study,^33,34^ Mediators of Atherosclerosis in South Asians Living in America,^35^ Multi-Ethnic Study of Atherosclerosis,^36^ Northern Manhattan Study,^37^ Prevent Pulmonary Fibrosis,^38^ Reasons for Geographic and Racial Differences in Stroke,^39^ Severe Asthma Research Program,^40^ Subpopulations and Intermediate Outcome Measures in COPD Study,^41^ and the Strong Heart Study.^42,43^ The study was approved by cohort-specific institutional review boards as well as the Columbia University institutional review board. Participants provided informed consent for COVID-19–related follow-up activities according to cohort-specific procedures, including verbal, remote, and written informed consent. Data access was governed by an Analysis Commons model to facilitate data sharing while maintaining confidentiality and aligning with cohort-specific data use agreements.^25^ This report includes participants who responded to the resilience question on C4R questionnaires completed between January 2021 and February 2023 (eFigure 1 in Supplement 1). This study follows the Strengthening the Reporting of Observational Studies in Epidemiology (STROBE) reporting guideline for cross-sectional studies. Details on each included cohort are provided in the eMethods in Supplement 1.

### Outcome

The primary outcome was self-reported resilience during the COVID-19 pandemic, assessed via a single item from the BRS: “I tend to bounce back quickly after hard times.”^44^ Although the BRS is a standardized 6-item questionnaire, only 1 item was included in the C4R survey to ensure consistent, efficient data collection across cohorts and to minimize participant burden. Previous research supports the validity of shortened resilience measures. For example, Vaishnavi et al^45^ found that a 2-item version of the Connor-Davidson Resilience Scale correlated strongly with the full scale (*r* = 0.78; *P* < .001), suggesting that carefully selected items for practical purposes may effectively represent the broader construct of resilience.^45^ For this study, participants rated their agreement on a 5-point scale. Responses were dichotomized as resilient (agree or strongly agree) or not resilient (neutral, disagree, or strongly disagree).^46^

### Exposures

Factors considered potentially associated with resilience were race and ethnicity, demographic variables, clinical and behavioral factors, and social determinants of health. Race and ethnicity were self-reported using US 2000 census^47^ categories: Hispanic, non-Hispanic Asian (further divided into East Asian and South Asian), non-Hispanic American Indian (hereafter, *American Indian*), non-Hispanic Black (hereafter, *Black*), and non-Hispanic White (hereafter, *White*).^25^ Region (Midwest, Middle Atlantic, New England, South, Southwest, or West) was also included. Demographic variables included sex and age at C4R survey completion. Finer stratifications of age (<45, 45-54, 55-64, 65-74, 75-84, or ≥85 years) was initially explored, but no significant differences were found. Thus, age was grouped as younger than 65 years, 65 to 74 years, 75 to 84 years, and 85 years or older for final analyses (eTable 1 in Supplement 1). Clinical and behavioral factors were assessed at the most recent prepandemic examination, including body mass index (BMI), smoking status (never, former, or current), hypertension (self-reported blood pressure reading of ≥140/90 mm Hg or use of antihypertensive medications), diabetes (self-reported fasting blood glucose ≥126 mg/dL [to convert to millimoles per liter, multiply by 0.0555] or use of hypoglycemic medications), COVID-19 vaccination status at C4R survey completion, and self-reported COVID-19 infection status at C4R survey completion. Social determinants of health assessed prior to the pandemic included educational attainment (<high school, high school, some college, or college graduate), marital status (single, married or living as married, widowed, divorced, or separated), employment status (employed or other than employed), health insurance (private insurance only [eg, employer-provided or individually purchased plans], public insurance only [eg, Medicare or Medicaid], both private and public insurance, no insurance, or unknown insurance type [reported having insurance but the specific type was not identified]), and consumer price index–adjusted annual household income (<$50 000, $50 000-100 000, or >$100 000).

### Statistical Analysis

Associations of self-reported resilience with potential correlates were examined using modified Poisson regression models. This approach was chosen to directly estimate prevalence ratios (PRs), which are more appropriate for common outcomes in cross-sectional studies. A minimally adjusted model included race and ethnicity, age, and sex. The fully adjusted model incorporated BMI, smoking status, hypertension, diabetes, COVID-19 vaccination status at questionnaire completion, infection status at questionnaire completion, region, and social determinants of health (education, marital status, employment status, health insurance, and income). Association modification by race and ethnicity was tested via multiplicative interaction terms and fully stratified models.

Missing data were minimal for most variables (<5%), with the exception of marital status (7.6%), household income (12.6%), and insurance status (9.4%) (Table 1). To account for missing covariate data, multiple imputation was implemented using the Multiple Imputation by Chained Equations (MICE)^48^ package in R software version 4.2.0 (R Project for Statistical Computing).^48^ Analyses were first conducted independently in 10 imputed datasets, then pooled using the PROC MIANALYZE procedure in SAS software version 9.4 (SAS Institute) under Rubin Rule.^49^ Complete-case analyses were included as secondary analyses. The timing of socioeconomic variable collection varied across cohorts, with some measures collected years before resilience assessment. To address potential bias from this temporal discordance, we conducted additional sensitivity analyses adjusting for the time between covariate measurement and resilience assessment. Time-to-measurement variables (in years) for marital status, employment status, insurance, and income were included in models, along with interactions terms to test whether associations with resilience varied by the timing of socioeconomic variable measurement.

**Table 1.** Sociodemographic Characteristics of Participants With Missingness, Stratified by Race/Ethnicity

Characteristic^a^	Participants, No. (%)							
	American Indian (n = 1185 [3.8%])	Black (n = 6724 [21.7%])	East Asian (n = 293 [0.9%])^a^	Hispanic (n = 6307 [20.3%])	South Asian (n = 565 [1.8%])^a^	White (n = 15 933 [51.3%])	Other (n = 38 [0.1%])	Total (N = 31 045 [100%])
Resilience^b^								
Disagree	394 (33.2)	1599 (23.8)	132 (45.1)	1556 (24.7)	138 (24.4)	4110 (25.8)	13 (34.2)	7942 (25.6)
Agree	791 (66.8)	5125 (76.2)	161 (54.9)	4751 (75.3)	427 (75.6)	11823 (74.2)	25 (65.8)	23 103 (74.4)
Sex								
Female	804 (67.8)	4399 (65.4)	155 (52.9)	4060 (64.4)	322 (57.0)	8915 (56.0)	17 (44.7)	18 672 (60.1)
Male	381 (32.2)	2325 (34.6)	138 (47.1)	2247 (35.6)	243 (43.0)	7016 (44.0)	21 (55.3)	12 371 (39.8)
Missing	0	0	0	0	0	2 (<0.1)	0	2 (<0.1)
Age, y								
<65	630 (53.2)	2045 (30.4)	15 (5.1)	3805 (60.3)	325 (57.5)	3909 (24.5)	17 (44.7)	10 746 (34.6)
65-74	177 (14.9)	2021 (30.1)	133 (45.4)	1552 (24.6)	170 (30.1)	4189 (26.3)	8 (21.1)	8250 (26.6)
75-84	226 (19.1)	1958 (29.1)	95 (32.4)	796 (12.6)	70 (12.4)	5542 (34.8)	11 (28.9)	8698 (28.0)
≥85	43 (3.6)	685 (10.2)	50 (17.1)	154 (2.4)	0	2270 (14.2)	2 (5.3)	3204 (10.3)
Missing	109 (9.2)	15 (0.2)	0	0	0	23 (0.1)	0	147 (0.5)
BMI								
<18.5	7 (0.6)	41 (0.6)	7 (2.4)	37 (0.6)	1 (0.2)	145 (0.9)	0	238 (0.8)
18.5-24.9	157 (13.2)	981 (14.6)	177 (60.4)	1036 (16.4)	222 (39.3)	4412 (27.7)	18 (47.4)	7003 (22.6)
25.0-29.9	351 (29.6)	2133 (31.7)	94 (32.1)	2494 (39.5)	251 (44.4)	5977 (37.5)	11 (28.9)	11 311 (36.4)
30.0-39.9	500 (42.2)	2821 (42.0)	15 (5.1)	2393 (37.9)	86 (15.2)	4289 (26.9)	8 (21.1)	10 112 (32.6)
≥40.0	169 (14.3)	739 (11.0)	0	343 (5.4)	5 (0.9)	608 (3.8)	0	1864 (6.0)
Missing	1 (0.1)	9 (0.1)	0	4 (0.1)	0	502 (3.2)	1 (2.6)	517 (1.7)
Smoking status								
Never	459 (38.7)	3477 (51.7)	215 (73.4)	3830 (60.7)	459 (81.2)	7094 (44.5)	23 (60.5)	15 557 (50.1)
Former	296 (25.0)	2134 (31.7)	72 (24.6)	1578 (25.0)	93 (16.5)	7368 (46.2)	6 (15.8)	11 547 (37.2)
Current	427 (36.0)	1050 (15.6)	6 (2.0)	873 (13.8)	13 (2.3)	1428 (9.0)	2 (5.3)	3799 (12.2)
Missing	3 (0.3)	63 (0.9)	0	26 (0.4)	0	43 (0.3)	7 (18.4)	142 (0.5)
Diabetes								
No	740 (62.4)	4763 (70.8)	227 (77.5)	4777 (75.7)	276 (48.8)	13256 (83.2)	29 (76.3)	24 068 (77.5)
Yes	442 (37.3)	1932 (28.7)	66 (22.5)	1530 (24.3)	289 (51.2)	2467 (15.5)	9 (23.7)	6735 (21.7)
Missing	3 (0.3)	29 (0.4)	0	0	0	210 (1.3)	0	242 (0.8)
Hypertension								
No	474 (40.0)	1642 (24.4)	122 (41.6)	3044 (48.3)	306 (54.2)	6409 (40.2)	22 (57.9)	12 019 (38.7)
Yes	711 (60.0)	5081 (75.6)	171 (58.4)	3263 (51.7)	259 (45.8)	9395 (59.0)	16 (42.1)	18 896 (60.9)
Missing	0	1 (<0.1)	0	0	0	129 (0.8)	0	130 (0.4)
Vaccine status at completion of C4R survey								
No	226 (19.1)	637 (9.5)	22 (7.5)	1047 (16.6)	181 (32.0)	2486 (15.6)	11 (28.9)	4610 (14.8)
Yes	900 (75.9)	5784 (86.0)	264 (90.1)	5161 (81.8)	383 (67.8)	13239 (83.1)	26 (68.4)	25 757 (83.0)
Missing	59 (5.0)	303 (4.5)	7 (2.4)	99 (1.6)	1 (0.2)	208 (1.3)	1 (2.6)	678 (2.2)
Infection status at completion of C4R survey								
No	724 (61.1)	6025 (89.6)	282 (96.2)	4350 (69.0)	534 (94.5)	14 254 (89.5)	31 (81.6)	26 200 (84.4)
Yes	461 (38.9)	682 (10.1)	11 (3.8)	1956 (31.0)	31 (5.5)	1640 (10.3)	6 (15.8)	4787 (15.4)
Missing	0	17 (0.3)	0	1	0	39 (0.2)	1 (2.6)	58 (0.2)
Education								
<High school	157 (13.2)	685 (10.2)	65 (22.2)	2275 (36.1)	6 (1.1)	522 (3.3)	1 (2.6)	3711 (12.0)
High school	514 (43.4)	1669 (24.8)	42 (14.3)	1533 (24.3)	16 (2.8)	2919 (18.3)	3 (7.9)	6696 (21.6)
Some college	291 (24.6)	1467 (21.8)	59 (20.1)	958 (15.2)	27 (4.8)	3103 (19.5)	5 (13.2)	5910 (19.0)
≥College	208 (17.6)	2790 (41.5)	127 (43.3)	1503 (23.8)	516 (91.3)	8530 (53.5)	23 (60.5)	13 697 (44.1)
Missing	15 (1.3)	113 (1.7)	0	38 (0.6)	0	859 (5.4)	6 (15.8)	1031 (3.3)
Marital status								
Single	264 (22.3)	750 (11.2)	4 (1.4)	1374 (21.8)	8 (1.4)	1190 (7.5)	4 (10.5)	3594 (11.6)
Married or living as married	601 (50.7)	3122 (46.4)	241 (82.3)	3427 (54.3)	525 (92.9)	10172 (63.8)	23 (60.5)	18 111 (58.3)
Widowed	74 (6.2)	792 (11.8)	32 (10.9)	85 (1.3)	20 (3.5)	1264 (7.9)	0	2267 (7.3)
Divorced or separated	232 (19.6)	1415 (21.0)	16 (5.5)	1393 (22.1)	12 (2.1)	1652 (10.4)	6 (15.8)	4726 (15.2)
Missing	14 (1.2)	645 (9.6)	0	28 (0.4)	0	1655 (10.4)	5 (13.2)	2347 (7.6)
Employment status								
Employed	256 (21.6)	3923 (58.3)	117 (39.9)	2969 (47.1)	416 (73.6)	7976 (50.1)	25 (65.8)	15 682 (50.5)
Other than employed	100 (8.4)	2666 (39.6)	176 (60.1)	3303 (52.4)	141 (25.0)	7360 (46.2)	8 (21.1)	13 754 (44.3)
Missing	829 (70.0)	135 (2.0)	0	35 (0.6)	8 (1.4)	597 (3.7)	5 (13.2)	1609 (5.2)
Health insurance								
No insurance	103 (8.7)	427 (6.4)	7 (2.4)	1525 (24.2)	33 (5.8)	277 (1.7)	5 (13.2)	2377 (7.7)
Private insurance only	134 (11.3)	1965 (29.2)	75 (25.6)	291 (4.6)	442 (78.2)	4258 (26.7)	16 (42.1)	7181 (23.1)
Public insurance only	37 (3.1)	599 (8.9)	107 (36.5)	299 (4.7)	48 (8.5)	770 (4.8)	5 (13.2)	1865 (6.0)
Private and public insurance	18 (1.5)	351 (5.2)	98 (33.4)	186 (2.9)	37 (6.5)	1016 (6.4)	1 (2.6)	1707 (5.5)
Unknown insurance type^c^	23 (1.9)	2999 (44.6)	6 (2.0)	3981 (63.1)	0	7985 (50.1)	7 (18.4)	15 001 (48.3)
Missing	870 (73.4)	383 (5.7)	0	25 (0.4)	5 (0.9)	1627 (10.2)	4 (10.5)	2914 (9.4)
Household income, $^d^								
<50 000	761 (64.2)	3376 (50.2)	188 (64.2)	4750 (75.3)	51 (9.0)	5044 (31.7)	4 (10.5)	14 174 (45.7)
50 000-100 000	249 (21.0)	1568 (23.3)	58 (19.8)	776 (12.3)	65 (11.5)	4074 (25.6)	5 (13.2)	6795 (21.9)
>100 000	48 (4.1)	1097 (16.3)	47 (16.0)	216 (3.4)	435 (77.0)	4298 (27.0)	8 (21.1)	6149 (19.8)
Missing	127 (10.7)	683 (10.2)	0	565 (9.0)	14 (2.5)	2517 (15.8)	21 (55.3)	3927 (12.6)
Region								
Middle Atlantic	8 (0.7)	838 (12.5)	3 (1.0)	1649 (26.1)	0	2254 (14.1)	8 (21.1)	4760 (15.3)
Midwest	581 (49.0)	652 (9.7)	118 (40.3)	995 (15.8)	232 (41.1)	3466 (21.8)	0	6044 (19.5)
New England	21 (1.8)	90 (1.3)	0	60 (1.0)	0	2606 (16.4)	17 (44.7)	2794 (9.0)
South Asian	2 (0.2)	4365 (64.9)	8 (2.7)	1676 (26.6)	0	5187 (32.6)	2 (5.3)	11240 (36.2)
Southwest	566 (47.8)	118 (1.8)	4 (1.4)	11 (0.2)	0	430 (2.7)	0	1129 (3.6)
West	7 (0.6)	603 (9.0)	159 (54.3)	1794 (28.4)	333 (58.9)	1561 (9.8)	2 (5.3)	4459 (14.4)
Missing	0	58 (0.9)	1 (0.3)	122 (1.9)	0	429 (2.7)	9 (23.7)	619 (2.0)

Abbreviations: BMI, body mass index (calculated as weight in kilograms divided by height in meters squared); C4R, Collaborative Cohort of Cohorts for COVID-19 Research.

^a^ Asian participants in cohorts other than Mediators of Atherosclerosis in South Asians Living in America or Multi-Ethnic Study of Atherosclerosis were dropped due to unclear race and ethnicity measurements and small sample sizes.

^b^ Wave 1 questionnaire resilience is favored when participants have both wave 1 and wave 2 measurements. We adopted the first record of resilience. Additionally, we combined the original strongly disagree, disagree, and neutral groups into the new disagree group and the original strongly agree and agree groups into the new agree group.

^c^ Participants have insurance, but the exact insurance type is unknown.

^d^ Income is standardized into 2020 dollars using customer price index (2020 = 258.811).

All analyses were conducted using SAS software. Statistical significance was defined as a 2-tailed α = .05. Data were initially analyzed from October 2023 to May 2024, with updated analyses performed from August 2024 to April 2025.

## Results

Participant characteristics are provided in Table 1 by self-reported race and ethnicity and in Table 2 by self-reported resilience. Of 31 043 participants in the imputed dataset, 10 828 (34.9%) were younger than 65 years, 18 672 (60.1%) were female, and 23 102 (74.4%) were classified as resilient. In the imputed sample, 1185 participants (3.8%) were American Indian, 6728 participants (21.7%) were Black, 6311 participants (20.3%) were Hispanic, 293 participants (0.9%) were East Asian, and 565 participants (1.8%) were South Asian, and 15 961 participants (51.4%) were White. The prevalence of self-reported resilience differed significantly across racial and ethnic groups, ranging from 76.2% for Black participants to 54.9% for East Asian participants (*P* < .001) (Table 1). Differences in self-reported resilience were also observed across BMI category, smoking status, hypertension, diabetes, COVID-19 vaccination status, COVID-19 infection status, education, marital status, employment status, health insurance, and income level (Table 2).

**Table 2.** Participant Characteristics by Self-Reported Resilience Status Using Multiple Imputed Data

Characteristic^a^	Participants, No. (%)		
	Response to “I tend to bounce back quickly after hard times”		Total
	Agree or strongly agree^b^	Neutral, disagree, or strongly disagree^b^	
Total	23 102 (74.4)	7941 (25.6)	31 043 (100)
Race and ethnicity^c^			
American Indian	791 (3.4)	394 (5.0)	1185 (3.8)
Black	5128 (22.2)	1600 (20.1)	6728 (21.7)
East Asian	161 (0.7)	132 (1.7)	293 (0.9)
Hispanic	4753 (20.6)	1558 (19.6)	6311 (20.3)
South Asian	427 (1.8)	138 (1.7)	565 (1.8)
White	11 842 (51.3)	4119 (51.9)	15 961 (51.4)
Age group, y			
<65	8084 (35.0)	2744 (34.6)	10 828 (34.9)
65-74	6190 (26.8)	2085 (26.3)	8275 (26.7)
75-84	6522 (28.2)	2204 (27.8)	8726 (28.1)
≥85	2306 (10.0)	907 (11.4)	3213 (10.4)
Sex			
Female	13 591 (58.8)	5081 (64.0)	18 672 (60.1)
Male	9511 (41.2)	2860 (36.0)	12 371 (39.9)
BMI			
<18.5	176 (0.8)	72 (0.9)	248 (0.8)
18.5-24.9	5223 (22.6)	1945 (24.5)	7168 (23.1)
25.0-29.9	8688 (37.6)	2790 (35.1)	11 478 (37.0)
30.0-39.9	7669 (33.2)	2575 (32.4)	10 244 (33.0)
≥40.0	1346 (5.8)	559 (7.0)	1905 (6.1)
Smoking status			
Never	11 591 (50.2)	4042 (50.9)	15 633 (50.4)
Former	8763 (37.9)	2831 (35.7)	11 594 (37.3)
Current	2748 (11.9)	1067 (13.4)	3815 (12.3)
Diabetes	4902 (21.2)	1866 (23.5)	6768 (21.8)
Hypertension	14 028 (60.7)	4914 (61.9)	18 942 (61.0)
Vaccinated at completion of C4R survey	19 616 (84.9)	6713 (84.5)	26 329 (84.8)
COVID-19 infection at completion of C4R survey	3589 (15.5)	1206 (15.2)	4795 (15.4)
Education			
<High school	2655 (11.5)	1161 (14.6)	3816 (12.3)
High school	5092 (22.0)	1861 (23.4)	6953 (22.4)
Some college	4540 (19.7)	1600 (20.1)	6140 (19.8)
≥College	10 815 (46.8)	3319 (41.8)	14 134 (45.5)
Marital status			
Single	2890 (12.5)	1106 (13.9)	3996 (12.9)
Married or living as married	14 628 (63.3)	4764 (60.0)	19 392 (62.5)
Widowed	1821 (7.9)	657 (8.3)	2478 (8.0)
Divorced or separated	3763 (16.3)	1413 (17.8)	5176 (16.7)
Employed	12 889 (55.8)	4018 (50.6)	16 907 (54.5)
Health insurance			
No insurance	2208 (9.6)	794 (10.0)	3002 (9.7)
Private insurance only	6084 (26.3)	1808 (22.8)	7892 (25.4)
Public insurance only	1438 (6.2)	644 (8.1)	2082 (6.7)
Private and public insurances	1322 (5.7)	472 (5.9)	1794 (5.8)
Unknown type of insurance^d^	12 050 (52.2)	4223 (53.2)	16 273 (52.4)
Household income, $^e^			
<50 000	11 845 (51.3)	4559 (57.4)	16 404 (52.8)
50 000-100 000	5804 (25.1)	1841 (23.2)	7645 (24.6)
>100 000	5453 (23.6)	1541 (19.4)	6994 (22.5)
Region			
Middle Atlantic	3577 (15.5)	1275 (16.1)	4852 (15.6)
Midwest	4615 (20.0)	1547 (19.5)	6162 (19.8)
New England	2057 (8.9)	880 (11.1)	2937 (9.5)
South	8660 (37.5)	2757 (34.7)	11 417 (36.8)
Southwest	829 (3.6)	309 (3.9)	1138 (3.7)
West	3364 (14.6)	1173 (14.8)	4537 (14.6)
Study			
ARIC	3260 (14.1)	849 (10.7)	4109 (13.2)
CARDIA	1353 (5.9)	445 (5.6)	1798 (5.8)
COPDGene	1896 (8.2)	472 (5.9)	2368 (7.6)
FHS	2209 (9.6)	907 (11.4)	3116 (10.0)
HCHS/SOL	4161 (18.0)	1205 (15.2)	5366 (17.3)
JHS	1250 (5.4)	330 (4.2)	1580 (5.1)
MASALA	427 (1.8)	138 (1.7)	565 (1.8)
MESA	1402 (6.1)	507 (6.4)	1909 (6.1)
NOMAS	317 (1.4)	297 (3.7)	614 (2.0)
PrePF	392 (1.7)	133 (1.7)	525 (1.7)
REGARDS	5076 (22.0)	2087 (26.3)	7163 (23.1)
SARP	168 (0.7)	84 (1.1)	252 (0.8)
SHS	760 (3.3)	379 (4.8)	1139 (3.7)
SPIROMICS	431 (1.9)	108 (1.4)	539 (1.7)

Abbreviations: ARIC, Atherosclerosis Risk in Communities; BMI, body mass index (calculated as weight in kilograms divided by height in meters squared); C4R, Collaborative Cohort of Cohorts for COVID-19 Research; CARDIA, Coronary Artery Risk Development in Young Adults; COPDGene, Genetic Epidemiology of COPD; FHS, Framingham Heart Study; HCHS/SOL, Hispanic Community Health Study/Study of Latinos; JHS, Jackson Heart Study; MASALA, Mediators of Atherosclerosis in South Asians Living in America; MESA, Multi-Ethnic Study of Atherosclerosis; NOMAS, Northern Manhattan Study; PrePF, Prevent Pulmonary Fibrosis; REGARDS; Reasons for Geographic and Racial Differences in Stroke; SARP, Severe Asthma Research Program; SHS, Strong Heart Study; SPIROMICS, Subpopulations and Intermediate Outcome Measures in COPD Study.

^a^ All counts and column percentages are mean values from 10 imputed datasets. 25 Of 310 450 imputed observations, 25 were deleted since East Asian participants were only from MESA and South Asian participants were only from MASALA.

^b^ Wave 1 questionnaire resilience is favored when participants have both wave 1 and wave 2 measurements. We adopted the first record of resilience.

^c^ Asian participants in cohorts other than MASALA or MESA were dropped due to unclear race and ethnicity measurements and small sample sizes.

^d^ Participants have insurance, but the exact insurance type is unknown.

^e^ Income is standardized into 2020 dollars using customer price index (2020 = 258.811).

### Factors Associated With Self-Reported Resilience

Factors associated with resilience are presented in Table 3. In the fully adjusted model and compared with White participants, American Indian participants had 10% lower prevalence of self-reported resilience (aPR, 0.90; 95% CI, 0.86-0.94), Black participants had 4% higher prevalence (aPR, 1.04; 95% CI, 1.02-1.06), Hispanic participants had 8% higher prevalence (aPR, 1.08; 95% CI, 1.06-1.11), and East Asian participants had 24% lower prevalence (aPR, 0.76; 95% CI, 0.68-0.84).

**Table 3.** Multivariable-Adjusted Associations With Self-Reported Resilience Using Multiple Imputed Data

Variable	Model 1		Model 2	
	PR (95% CI)	*P* value	PR (95% CI)	*P* value
Race and ethnicity				
American Indian	0.90 (0.87-0.94)	<.001	0.90 (0.86-0.94)	<.001
Black	1.03 (1.01-1.05)	<.001	1.04 (1.02-1.06)	<.001
East Asian	0.74 (0.67-0.82)	<.001	0.76 (0.68-0.84)	<.001
Hispanic	1.02 (1.00-1.03)	.09	1.08 (1.06-1.11)	<.001
South Asian	1.01 (0.97-1.06)	.57	0.96 (0.91-1.01)	.10
White	1 [Reference]	NA	1 [Reference]	NA
Age group, y				
<64	1 [Reference]	NA	1 [Reference]	NA
65-74	1.00 (0.98-1.02)	.88	1.02 (1.00-1.04)	.08
75-84	1.00 (0.98-1.02)	.91	1.01 (0.99-1.04)	.21
Age >85	0.96 (0.94-0.99)	.003	0.98 (0.95-1.01)	.13
Sex				
Male	1 [Reference]	NA	1 [Reference]	NA
Female	0.95 (0.93-0.96)	<.001	0.96 (0.95-0.97)	<.001
BMI				
<18.5	NA	NA	0.99 (0.91-1.08)	.83
18.5-24.9	1 [Reference]	NA	1 [Reference]	NA
25.0-29.9	NA	NA	1.03 (1.01-1.05)	.003
30.0-39.9	NA	NA	1.02 (1.00-1.04)	.02
≥40.0	NA	NA	0.98 (0.95-1.01)	.19
Smoking status				
Never	1 [Reference]	NA	1 [Reference]	NA
Former	NA	NA	1.02 (1.01-1.04)	.002
Current	NA	NA	0.99 (0.97-1.01)	.48
Diabetes^a^	NA	NA	0.97 (0.96-0.99)	.002
Hypertension^a^	NA	NA	0.99 (0.97-1.00)	.10
Vaccinated at completion of C4R survey^a^	NA	NA	0.99 (0.97-1.01)	.49
COVID-19 infection at completion of C4R survey^a^	NA	NA	1.01 (0.99-1.03)	.47
Education				
<High school	NA	NA	1 [Reference]	NA
High school	NA	NA	1.07 (1.04-1.09)	<.001
Some college	NA	NA	1.07 (1.05-1.10)	<.001
≥College	NA	NA	1.09 (1.07-1.12)	<.001
Marital status				
Single	NA	NA	0.97 (0.95-0.99)	.02
Married or living as married	NA	NA	1 [Reference]	NA
Widowed	NA	NA	1.03 (1.00-1.06)	.03
Divorced or separated	NA	NA	0.98 (0.96-1.00)	.05
Employment status				
Employed	NA	NA	1 [Reference]	NA
Other than employed	NA	NA	0.96 (0.94-0.97)	<.001
Health insurance				
No insurance	NA	NA	1.04 (1.00-1.08)	.08
Public insurance only	NA	NA	1 [Reference]	NA
Private insurance only	NA	NA	1.07 (1.03-1.10)	<.001
Private and public insurances	NA	NA	1.05 (1.00-1.09)	.04
Unknown type of insurance	NA	NA	1.02 (0.99-1.06)	.20
Income, $				
<50 000	NA	NA	1 [Reference]	NA
50 000-100 000	NA	NA	1.03 (1.01-1.04)	.01
>100 000	NA	NA	1.05 (1.03-1.07)	<.001
Region				
Middle Atlantic	NA	NA	0.98 (0.96-1.01)	.18
Midwest	NA	NA	1 [Reference]	NA
New England	NA	NA	0.90 (0.88-0.93)	<.001
South	NA	NA	0.99 (0.97-1.01)	.37
Southwest	NA	NA	1.03 (0.99-1.08)	.12
West	NA	NA	0.98 (0.96-1.01)	.15

Abbreviations: BMI, body mass index (calculated as weight in kilograms divided by height in meters squared); C4R, Collaborative Cohort of Cohorts for COVID-19 Research; NA, not applicable.

Higher prevalences of resilience were observed in participants with a high school degree (aPR, 1.07; 95% CI, 1.04-1.09), some college (aPR, 1.07; 95% CI, 1.05-1.10), or a college degree (aPR, 1.09; 95% CI, 1.07-1.12), compared with those with less than a high school education. Single participants reported lower prevalence of resilience compared with married or living as married participants (aPR, 0.97; 95% CI, 0.95-0.99). Not being employed was associated with lower prevalence of resilience (aPR, 0.96; 95% CI, 0.94-0.97).

Compared with participants with public insurance only, participants with private insurance only reported 7% higher prevalence of resilience (aPR, 1.07; 95% CI, 1.03-1.10). Compared with participants earning less than $50 000 annually, participants earning $50 000 to $100 000 (aPR, 1.03; 95% CI, 1.01-1.04) or more than $100 000 annually (aPR, 1.05; 95% CI, 1.03-1.07) had higher prevalence of resilience. Regional differences in self-reported resilience were observed. Compared with the Midwest region, participants from New England reported 10% lower prevalence of resilience (aPR, 0.90; 95% CI, 0.88-0.93).

### Analyses Stratified by Race and Ethnicity

**Figure 1.**
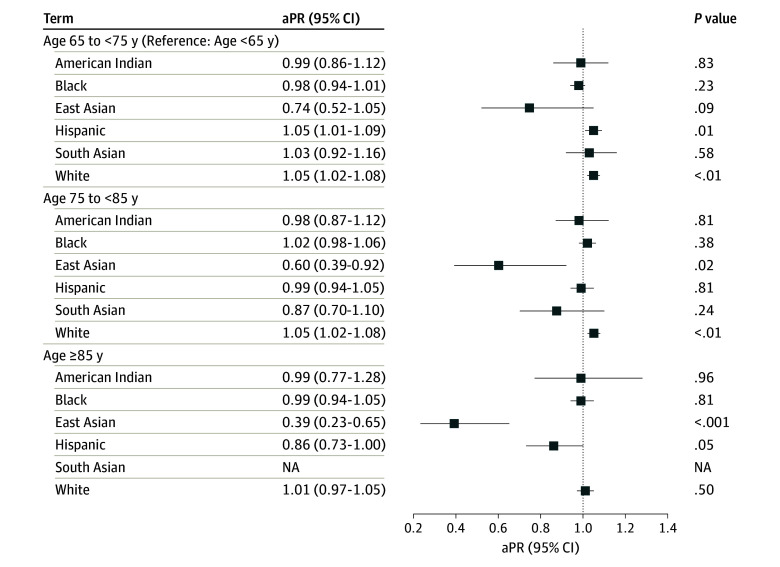
Associations of Age Groups With Self-Reported Resilience by Race and Ethnicity

**Figure 2.**
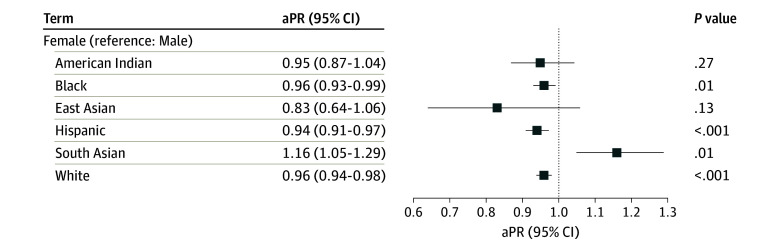
Associations of Sex With Self-Reported Resilience by Race and Ethnicity

Results were similar in models stratified by race and ethnicity, although there was evidence to suggest association modification for several factors. A significant interaction observed between race and ethnicity and age (*P* for interaction < .001) indicated that age-related patterns of resilience differed by groups (Figure 1). East Asian participants demonstrated a notable decline in resilience with age, with lower resilience among those aged 75 to 84 years (aPR, 0.60; 95% CI, 0.39-0.92) and 85 years or older (aPR, 0.39; 95% CI, 0.23-0.65) compared with those younger than 65 years. In contrast, no similar age-related decline were observed in American Indian, Black, or White participants. In fact, resilience were slightly higher among Black (aPR, 1.05; 95% CI, 1.01-1.09) and White (aPR, 1.05; 95% CI, 1.02-1.08) participants aged 75 to 84 years. Compared with males, females reported lower prevalence of resilience among Black (aPR, 0.96; 95% CI, 0.93-0.99), Hispanic (aPR, 0.94; 95% CI, 0.91-0.97), and White (aPR, 0.96; 95% CI, 0.94-0.98) participants and higher prevalence of resilience among South Asian participants (aPR, 1.16; 95% CI, 1.05-1.29) (*P* for interaction = .02) (Figure 2). Hypertension was associated with lower resilience in Hispanic participants only (aPR, 0.95; 95% CI, 0.92-0.98; *P* for interaction < .001) (eFigure 2 in Supplement 1). Regional variation was significant (*P* for interaction < .001). Compared with the Midwest, while Hispanic individuals in the South and West had higher resilience, while White participants in these regions had lower resilience. American Indian participants in the South demonstrated significantly greater resilience (eFigure 3 in Supplement 1).

Health insurance modified associations with resilience (*P* for modification < .001) (eFigure 4 in Supplement 1). Among Hispanic participants, no insurance was associated with higher prevalence of resilience (aPR, 1.24; 95% CI, 1.11-1.39), while opposite associations were seen in other racial and ethnical groups. BMI also was associated with resilience (eFigure 5 in Supplement 1). Notably, obesity was associated with lower resilience among American Indian participants (aPR, 0.86; 95% CI, 0.77-0.96;) but higher resilience among White participants (aPR, 1.04; 95% CI, 1.01-1.07). Diabetes interacted significantly with race and ethnicity (*P* for interaction = .03), with American Indian participants showing the strongest negative association (aPR, 0.91; 95% CI, 0.82-0.99) (eFigure 6 in Supplement 1). Furthermore, marital status was only significant among Black participants, among whom single participants had lower prevalence of resilience (aPR, 0.92; 95% CI, 0.88-0.97; *P* = .01) (eFigure 7 in Supplement 1). No significant interactions were observed for education, income, smoking status, COVID-19 vaccination status, or infection status.

### Complete Case and Sensitivity Analyses

Participant characteristics for complete cases were similar to the primary (imputed) analysis sample (eTable 2 in Supplement 1). Sensitivity analyses comparing complete case analysis for 23 154 participants with our multiple imputation approach showed consistent patterns of association (eTable 3 and eFigure 8 in Supplement 1). In sensitivity analyses adjusting for the time between covariate measurement and resilience assessment, patterns of association remained largely consistent. While the time-to-income variables were statistically significant, their effect sizes were minimal. None of the interactions between time-to-measurement variables and social determinants of health variables reached statistical significance (eTable 4 in Supplement 1).

## Discussion

In this cross-sectional study of a nationwide meta-cohort of US adults, self-reported resilience was remarkably high but differed by sociodemographic and clinical factors. Greater self-reported resilience was observed among Black and Hispanic participants, individuals with higher education, married individuals, and individuals with higher income, whereas being female or unemployed was associated with lower resilience.

These findings should be interpreted in the context of the COVID-19 pandemic, which created an unprecedented global crisis that intensified existing social, economic, and health disparities.^50^ The strain on health care systems, widespread job losses, and disruptions to daily life likely had profound impacts on individuals’ perception of their ability to cope with and bounce back from adversity.^14,51,52^ In this context, the observed differences in self-reported resilience show a complex interplay between systemic inequities and adaptations.^53,54^ For example, the lower prevalence of resilience among East Asian participants may relate to increased discrimination and stigma during the pandemic.^55^ Similarly, lower resilience among persons with lower education and income may reflect disproportionate economic hardship and limited resource access.^56,57^

The high prevalence of self-reported resilience among Black and Hispanic participants, despite the well-documented health and social inequities faced by these communities,^58^ may indicate the complex nature of resilience. This finding, in particular, shows the importance of considering resilience as a dynamic process shaped by the interaction between individuals and their environment.^59,60^ Resilience in marginalized populations reflects collective strengths, resources, and coping strategies developed in response to historical and ongoing adversity.^61,62^ These collective strengths, such as strong community networks, religious engagement and spirituality, extended family systems, and cultural practices, may foster resilience. Recognizing and building on theses community-level assets is essential, alongside addressing structural inequities.

Our findings align with previous research that used multi-item resilience measures and similarly found positive associations of resilience with education, income, and social support.^11,63,64^ Our study extends this knowledge, examining resilience specifically during the COVID-19 pandemic across a large cohort spanning multiple US regions. Research during the pandemic has found varied responses, with some populations showing significant psychological distress while others demonstrated remarkable adaptability despite ongoing stressors.^11^ Our findings contribute to this emerging literature by identifying specific factors associated with resilience during crisis that can inform targeted interventions for vulnerable groups in future public health emergencies. The persistent racial and ethnic differences in self-reported resilience after adjustment shows the need to identify and address unique challenges and resources that shape resilience in various populations, potentially leading to more effective interventions and policies. Other factors, such as varied interpretations of resilience across cultures, community contexts, and life-course experiences, also influence individuals’ ability to cope with adversity.^65,66^

Resilience is complex and has been defined and operationalized in various ways. It is often described as the ability to bounce back or recover from adversity, stress, or trauma.^1,59^ However, there is ongoing debate about the precise definition and measurement of resilience. Some researchers conceptualize resilience as a trait; others view it as a dynamic and evolving process.^67,68,69^ Findings of this study suggest resilience may not be solely an individual trait but, rather, a product of the complex interplay among individuals, their environment, and their current sociopolitical context, in this case, during the COVID-19 pandemic. The temporal nature of resilience development remains unclear, such as whether it responds rapidly to events like the pandemic or evolves more slowly over time.

Although reports of resilience in this study may reflect individuals’ ability to cope with and adapt to the pandemic, resilience levels and associated factors could differ in other contexts or noncrisis times. Resilience could also be conceptualized as healthy vs unhealthy resilience. Healthy resilience promotes overall well-being and growth, whereas unhealthy resilience relies on short-term coping mechanisms that ultimately may have detrimental effects on mental and physical health.^70,71^ Understanding this distinction requires consideration of both immediate coping ability and long-term outcomes. Our cross-sectional design, based on a single assessment during the pandemic, limits the ability to distinguish between short-term temporary coping and more enduring resilience processes that support long-term well-being.

Modification analyses revealed important nuances across racial and ethnic groups. Hypertension was associated with lower resilience only among Hispanic participants, suggesting possible cultural differences in how chronic conditions impact resilience. Similarly, while private insurance was associated with more resilience among most groups, patterns differed among Hispanic participants, pointing to potential differences in how health system engagement influences resilience across cultural contexts. These interactions demonstrate that resilience resources and vulnerabilities are not uniform across populations; therefore, culturally tailored approaches are needed to support resilience during crisis situations.

The findings of this study have important implications for interventions and policies aimed at promoting resilience in future crises. Whereas most participants in our study reported being resilient during the pandemic, it is unclear whether this resilience was healthy or unhealthy. Distinguishing between these different types of resilience requires validated multidimensional measures and longitudinal studies that examine long-term outcomes. Notably, the development of resilience is not solely an individual responsibility, it is also influenced by the broader social, economic, and political contexts. While our focus on individual-level social determinants provides important insights, structural and community-level factors, such as neighborhood characteristics, community resources, institutional racism, and historical inequities, likely play substantial roles but were not directly measured in the study.

Nevertheless, the observed socioeconomic disparities in resilience show the associations of systemic inequities and underinvestment in communities with individuals’ capacity to cope with adversity. Therefore, efforts to promote resilience must address structural inequities and social determinants of health through policies that ensure equitable access to health care, education, and economic opportunities and interventions that build community-level resilience through social support, collective action, and advocacy.^72,73^ Although individual-level interventions, such as teaching coping skills and stress management techniques, are beneficial, a comprehensive approach that recognizes the complex interplay among individual, community, and societal factors is necessary to foster resilience and promote health equity.^60,73,74^ It is important to note that the higher resilience observed among Black and Hispanic participants may reflect unmeasured community supports, such as strong social networks, cultural practices, or community organizations, which warrant further understanding.

Therefore, efforts to promote resilience should address not only individual-level factors but also select social determinants of health that may constrain or enable resilience in different populations.^75^ Recognizing the social and economic underpinnings of resilience is essential for designing interventions that promote health equity.

### Limitations

This study has several limitations. Although the sample was large and diverse, it was not nationally representative. Some additional factors related to resilience were not captured in this study, such as personality traits, coping strategies, and community-level factors. The cross-sectional design precludes causal inference and prevents assessment of pandemic-related changes in resilience. Timing of covariates measurements varied across cohorts, with some data collected years before the pandemic. However, sensitivity analyses adjusting for timing suggest this did not substantially bias our results.

A key limitation is our use of a single-item measure from the BRS.^44^ Although this allowed a snapshot of each participant’s perceived ability to bounce back from adversity and contributed to minimizing participant burden in the large-scale data collection across all 14 large cohorts during the pandemic, we recognize that although the BRS primarily measures a unidimensional construct focused on recovery from adversity,^44^ a single item may not capture all relevant aspects of this construct. Single-item measures have shown utility in large epidemiological studies where brevity is essential,^76^ and the specific item we used captured the core conceptual element of resilience—the ability to recover from adversity. While single-item measures typically demonstrate lower reliability compared with multi-item scales,^77^ our large sample size helps mitigate concerns about measurement error. The selected item directly captures the core theoretical concept of resilience as recovery from adversity, which is identified as the central construct measured by the BRS.^44,77^ Nevertheless, we acknowledge that our single-item approach may limit precision in measuring this important construct.

Furthermore, self-reported resilience introduces potential biases in how participants perceive and report their ability to bounce back from adversity. Cultural differences in interoperating resilience, social desirability, and varying perceptions of what it means to bounce back quickly may influence responses. Future research should incorporate objective measures of resilience, behavioral assessments, longitudinal designs, and mixed-methods approaches to triangulate findings. Additionally, some subgroup analysis were based on small sample sizes, leading to wider CIs and greater uncertainty. These estimates should be interpreted with caution.

## Conclusions

This cross-sectional study provided important insights into factors associated with self-reported resilience during the COVID-19 pandemic in a large, diverse US sample. Our findings highlight significant racial and ethnic differences in self-reported resilience and underscore the importance of social and structural factors. Higher education, income, and social support were consistently associated with greater resilience across racial and ethnic groups, highlighting the need for policies and interventions that promote access to these resources. The partial mediation of racial and ethnic disparities by social determinants of health further emphasizes the role of systematic inequities and the need for equity-focused approaches to promoting resilience in the face of adversity.

As the world continues to grapple with the impacts of the COVID-19 pandemic and other crises, understanding and promoting healthy resilience is important. The findings of this study illustrate the complex interplay of individual, social, and structural factors that shape resilience and emphasize the need for strategies that move beyond individual-level interventions to address broader systemic inequities. Building a more resilient, equitable, and just society requires not only supporting individuals but also dismantling the structural barriers that create vulnerability.

## Notes

Supplement 1.
eMethods.
**eFigure 1.** CONSORT Diagram of Participants Included in the Analyses **eTable 1.** Association Between Finer Stratification of Age Group and Self-reported Resilience a **eFigure 2.** Forest plot of associations of hypertension with self-reported resilience by race and ethnicity **eFigure 3.** Forest plot of associations of region with self-reported resilience by race and ethnicity **eFigure 4.** Forest plot of associations of insurance with self-reported resilience **eFigure 5.** Forest plot of associations of BMI categories with self-reported resilience by race and ethnicity **eFigure 6.** Forest plot of associations of diabetes with self-reported resilience by race and ethnicity **eFigure 7.** Forest plot of associations of marital status with self-reported resilience by race and ethnicity **eTable 2.** Participant Characteristics by Self-reported Resilience Status Using Complete Case Data **eTable 3.** Multivariable-adjusted Associations with Self-reported Resilience Using Complete Case Data **eFigure 8.** Forest plot showing correlates of self-reported resilience, comparing results from the multiple imputed **eTable 4.** Multivariable-adjusted associations with self-reported resilience, adjusting for the time between covariate measurement and resilience assessment
eReferences.

Supplement 2.
Data Sharing Statement
