# Supplementary material for: Self-Reported Resilience During the COVID-19 Pandemic
Source: JAMA Netw Open. 2025 Jul 16;8(7):e2520360. doi: 10.1001/jamanetworkopen.2025.20360 (PMC12268485; doi:10.1001/jamanetworkopen.2025.20360)
Supplement: Supplement 1. — eMethods. eFigure 1. CONSORT Diagram of Participants Included in the Analyses eTable 1. Association Between Finer Stratification of Age Group and Self-reported Resilience a eFigure 2. Forest plot of associations of hypertension with self-reported resilience by race and ethnicity eFigure 3. Forest plot of associations of region with self-reported resilience by race and ethnicity eFigure 4. Forest plot of associations of insurance with self-reported resilience eFigure 5. Forest plot of associations of BMI categories with self-reported resilience by race and ethnicity eFigure 6. Forest plot of associations of diabetes with self-reported resilience by race and ethnicity eFigure 7. Forest plot of associations of marital status with self-reported resilience by race and ethnicity eTable 2. Participant Characteristics by Self-reported Resilience Status Using Complete Case Data eTable 3. Multivariable-adjusted Associations with Self-reported Resilience Using Complete Case Data eFigure 8. Forest plot showing correlates of self-reported resilience, comparing results from the multiple imputed eTable 4. Multivariable-adjusted associations with self-reported resilience, adjusting for the time between covariate measurement and resilience assessment eReferences. [file jamanetwopen-e2520360-s001.pdf]

Supplemental Online Content

Ogungbe O, Wang T, Balte PP, et al. Self-reported resilience during the COVID-19 pandemic. *JAMA Netw Open*. 2025;8(7):e2520360. doi:10.1001/jamanetworkopen.2025.20360

eMethods.

eFigure 1. CONSORT Diagram of Participants Included in the Analyses

eTable 1. Association Between Finer Stratification of Age Group and Self-reported Resilience a

eFigure 2. Forest plot of associations of hypertension with self-reported resilience by race and ethnicity

eFigure 3. Forest plot of associations of region with self-reported resilience by race and ethnicity

eFigure 4. Forest plot of associations of insurance with self-reported resilience

eFigure 5. Forest plot of associations of BMI categories with self-reported resilience by race and ethnicity

eFigure 6. Forest plot of associations of diabetes with self-reported resilience by race and ethnicity

eFigure 7. Forest plot of associations of marital status with self-reported resilience by race and ethnicity

eTable 2. Participant Characteristics by Self-reported Resilience Status Using Complete Case Data

eTable 3. Multivariable-adjusted Associations with Self-reported Resilience Using Complete Case Data

eFigure 8. Forest plot showing correlates of self-reported resilience, comparing results from the multiple imputed

eTable 4. Multivariable-adjusted associations with self-reported resilience, adjusting for the time between covariate measurement and resilience assessment

eReferences.

This supplemental material has been provided by the authors to give readers additional information about their work.

© 2025 Ogungbe O et al. *JAMA Network Open*.

Supplementary Methods

Cohort Descriptions

Atherosclerosis Risk in Communities (ARIC)[1]: The ARIC study began in the mid 1980s with initial aims for its cohort component being to describe the presence of subclinical atherosclerosis (mainly via carotid ultrasound), the progression of atherosclerosis to clinical cardiovascular disease (CVD), and the association of novel risk factors with CVD. ARIC recruited its cohort of 15,792 men and women aged 45-64 in 1987-89 from four communities: Forsyth County, NC; Jackson, MS; suburban Minneapolis, MN; and Washington County, MD. The investigators used probability sampling to obtain a community wide sample, exclusively sampling African Americans in Jackson and oversampling African Americans in Forsyth County. ARIC conducted a baseline examination of cohort participants and up to eight subsequent examinations prior to the pandemic, with additional follow-up ongoing; performed annual or semi-annual telephone follow-up interviews; and throughout has identified and validated incident CVD and other outcomes, particularly cognitive decline in recent years.

Coronary Artery Risk Development in Young Adults (CARDIA)[2]: CARDIA is a study examining the development and determinants of clinical and subclinical CVD and their risk factors. It began in 1985-1986 with a cohort of 5115 Black and White men and women aged 18-30 years. The participants were selected so that there would be approximately the same number of people in subgroups of race (Black and White), gender (women and men), education (high school or less and more than high school) and age (18-24 and 25-30 years) in each of 4 field centers: Birmingham, AL; Chicago, IL; Minneapolis, MN; and Oakland, CA. These same participants were asked to participate in follow-up examinations during 1987-1988 (Year 2), 1990-1991 (Year 5), 1992-1993 (Year 7), 1995-1996 (Year 10), 2000-2001 (Year 15), 2005-2006 (Year 20), 2010-2011 (Year 25), 2015-2016 (Year 30), and 2020-2022 (Year 35). A majority of the group has been examined at each of the follow-up examinations (91%, 86%, 81%, 79%, 74%, 72%, 72%, 71%, and 67% [despite the impact of the COVID-19 pandemic on Year 35], respectively). While the specific aims of each examination have varied, data have been collected on a variety of factors believed to be related to heart disease. These include conditions

© 2025 Ogungbe O et al. *JAMA Network Open*.

with clear links to heart disease such as blood pressure, cholesterol and other lipids, and glucose. Data have also been collected on physical measurements such as weight and body composition as well as lifestyle factors such as dietary and exercise patterns, substance use (tobacco and alcohol), behavioral and psychological variables, medical and family history, and other chemistries (e.g., insulin). In addition, subclinical atherosclerosis has been measured via echocardiography during Years 5, 10, 25, and 30, a chest CT scan during Years 15, 20, 25, and 35, an abdominal CT scan during Years 25 and 35, and carotid ultrasound during Year 20. A brain MRI was performed on a subset of participants at Years 25, 30, and 35. The CARDIA cohort, born between 1955 and 1968, has been influenced substantially by the obesity epidemic at ages younger than participants in other established NHLBI cohorts. Further investigation of the mechanisms linking obesity to derangements in cardiovascular structure and function and the etiology of clinical events promises to generate important new knowledge to inform health promotion and disease prevention efforts.

Genetic Epidemiology of COPD (COPDGene)[3]: COPDGene is a non-interventional, multicenter, longitudinal, case-control study at 21 US sites of smokers with a ≥10 pack-year history with and without COPD and healthy never smokers. The goal was to characterize disease-related phenotypes and explore associations with susceptibility genes. COPDGene research participants were extensively phenotyped with the use of comprehensive symptom and comorbidity questionnaires, spirometry, chest CT scans, and genetic and biomarker profiling. The study enrolled 10,198 participants. COPDGene has had 3 exams that include spirometry, diffusing capacity, lung CT scans and other measures; its current exam is ongoing. COPDGene examines the influence of age, sex, and race on the natural history of COPD, and the impact of comorbid conditions, chronic bronchitis, exacerbations, and asthma/COPD overlap.

Framingham Heart Study (FHS):[4] FHS was initiated in 1948. Researchers recruited 5,209 men and women between the ages of 30 and 62 from the town of Framingham, Massachusetts, and began the first round of extensive physical examinations and lifestyle interviews that they would later analyze for common patterns related to CVD development. Since 1948, the subjects have returned to the study every two years for an examination consisting of a detailed medical history, physical examination, and laboratory tests, and in 1971, the study enrolled a second-generation

© 2025 Ogungbe O et al. *JAMA Network Open*.

cohort – 5,124 of the original participants' adult children and their spouses – to participate in similar examinations. The second examination of the Offspring cohort occurred eight years after the first examination, and subsequent examinations have occurred approximately every four years thereafter. In April 2002 the Study entered a new phase: the enrollment of a third generation of participants, the grandchildren of the original cohort. The first examination of the Third Generation Study was completed in July 2005 and involved 4,095 participants. Thus, the FHS has evolved into a prospective, community-based, three generation family study. In addition to research studies focused on risk factors, subclinical CVD and clinically apparent CVD, Framingham investigators have also collaborated with leading researchers from around the country and throughout the world on projects involving some of the major chronic illnesses in men and women, including dementia, osteoporosis and arthritis, nutritional deficiencies, eye diseases, hearing disorders, and chronic obstructive lung disease.

Hispanic Community Health Study/Study of Latinos (HCHS/SOL)[5–7]: HCHS/SOL is an ongoing population based prospective cohort study of 16,415 community dwelling Hispanic/Latino adults aged 18-74 years at baseline, recruited from four urban field centers with large populations of Hispanics/ Latinos (Bronx, NY; Chicago, IL; Miami, FL; and San Diego, CA). A two-stage area probability sample of households was selected, with stratification and over-sampling at each stage to ensure a diverse and representative sample.[5] Participants self-identified as Hispanic/Latino and of Cuban, Dominican, Mexican, Puerto Rican, Central American, South American, or other/more than one heritage. Study participants underwent an extensive clinic exam and assessments to determine baseline risk factors (2008-2011),[6] and annual telephone follow-up interviews for ascertainment of cardiovascular and pulmonary events. A second clinic visit was conducted in 2014-2017, and a third clinic visit was recently completed (2020-2024). The overall retention rate as of February 2023 was 81%. The primary goals of the HCHS/SOL are to describe: (1) the prevalence and incidence of cardiovascular, pulmonary, and other major chronic conditions (2) the risk and/or protective factors associated with these conditions; and (3) the relationships between the initial sociodemographic and health profiles and future health events in the target population. The study to date has revealed a high prevalence of cardiovascular risk factors, with significant variability by Hispanic/Latino heritage and sociodemographic factors such as income and time in the United States.[7]

© 2025 Ogungbe O et al. *JAMA Network Open*.

Jackson Heart Study (JHS)[8–10]: The JHS is a community-based cohort study evaluating risk factors for cardiovascular and related diseases among adult African Americans residing in the three counties (Hinds, Madison, and Rankin) that make up the Jackson, Mississippi metropolitan area. Data and biologic materials have been collected from 5,306 participants, including a nested family cohort of 1,498 members of 264 families. The age at enrollment for the unrelated cohort was 35-84 years; the family cohort included related individuals >21 years old. Participants have provided extensive medical and psychosocial histories and had an array of physical and biochemical measurements and diagnostic procedures during a baseline examination (2000-2004) and two follow-up examinations (2005-2008 and 2009-2012). Samples for genomic DNA were collected during the first two examinations. Annual follow-up interviews and cohort surveillance of cardiovascular events and mortality are continuing and a fourth examination is in progress.

Mediators of Atherosclerosis in South Asians Living in America (MASALA) study[11,12]: South Asians comprise almost one-quarter of the world's population and are the second fastest growing ethnic group in the US. The MASALA Study is a prospective cohort of South Asians called the MASALA study, which is closely tied to the Multi-Ethnic Study of Atherosclerosis (MESA), for valid cross-ethnic comparisons.[11] MASALA enrolled 906 South Asians in 2010-2013 and then added a new wave of 258 South Asian participants from 2017-2018, for a full cohort size of 1,164.[12] The original MASALA cohort has been followed for approximately 8.5 years, and completed a second clinical exam in early 2018. A third MASALA clinical exam is planned for 2022-2024. 75 papers have been published from MASALA to date, and the findings clearly show that the US South Asian population has a distinct phenotype compared to the other four race/ethnic groups studied in MESA. Major findings have included a higher prevalence of diabetes, ectopic adiposity and coronary artery calcium compared to MESA. The MASALA study findings have influenced guidelines for diabetes screening, lipid management, and raised awareness of South Asian CVD risk. MASALA is filling a large gap in scientific knowledge about CVD in a large, growing Asian American subgroup.

© 2025 Ogungbe O et al. *JAMA Network Open*.

Multi-Ethnic Study of Atherosclerosis (MESA)[13]: MESA is a study of the characteristics of subclinical CVD (disease detected non-invasively before it has produced clinical signs and symptoms) and the risk factors that predict progression to clinically overt cardiovascular disease or progression of the subclinical disease. MESA researchers study a diverse, population-based sample of 6,814 men and women aged 45-84 without known clinical cardiovascular disease. Thirty-eight percent of the recruited participants are white, 28 percent African-American, 22 percent Hispanic, and 12 percent of Chinese descent. Participants were recruited from six field centers across the United States: Wake Forest University, Columbia University, Johns Hopkins University, University of Minnesota, Northwestern University and University of California – Los Angeles. At baseline, each participant received an extensive physical exam and determination of coronary artery calcification, ventricular mass and function, flow-mediated endothelial vasodilation, carotid intimal-medial wall thickness and presence of echogenic lucencies in the carotid artery, lower extremity vascular insufficiency, arterial wave forms, electrocardiographic (ECG) measures, standard coronary risk factors, sociodemographic factors, lifestyle factors, and psychosocial factors. Selected repetition of subclinical disease measures and risk factors at follow-up visits allows study of the progression of disease. Blood samples have been assayed for putative biochemical and genetic risk factors and stored for case-control studies. Participants are being followed for identification and characterization of cardiovascular disease events, including acute myocardial infarction and other forms of coronary heart disease (CHD), stroke, and heart failure; for CVD interventions; and for mortality. The first examination took place over two years, from July 2000 – July 2002. It has been followed by six examination periods that were 17-20 months in length. Participants have been contacted every 9 to 12 months throughout the study to assess clinical morbidity and mortality.

Northern Manhattan Study (NOMAS)[14]: NOMAS began in 1993 as a population-based incidence and case-control study. In 1998 (cycle 2) the study evolved into a prospective cohort study of 3,298 stroke-free, tri-ethnic, community subjects followed annually to detect stroke, MI, and death. Starting in 2003 (cycle 3), subclinical measures (brain MRI & carotid ultrasound) and the first complete neuropsychological (NP) battery were collected on 1290 members (MRI cohort). The project has remained productive through subsequent cycles. As the cohort aged, the specific aims grew to include not only vascular determinants of stroke but also cognitive decline,

© 2025 Ogungbe O et al. *JAMA Network Open*.

mild cognitive impairment (MCI) and dementia. NOMAS participates in collaborative studies on genetics, stroke, MRI markers, Alzheimer Disease and neurodegenerative diseases. One of the major interests of the study has been the exploration of inflammatory and infectious contributors to stroke risk, subclinical atherosclerotic and cerebrovascular disease, and cognitive decline. The NOMAS community cohort of 3,298 subjects was assembled from a population-based, random sample based on the following criteria: (1) resident of at least 3 months of Northern Manhattan; (2) randomly derived from a household with a telephone; (3) age 40 or older at baseline (changed to age 55 in 1998); and (4) no history of stroke. The 1,290 subjects in the MRI cohort (median age 70 at MRI; 60% women, 15% non-Hispanic White, 17% non-Hispanic Black, 66% Hispanic, 2% Other) were evaluated with a standardized brain MRI and NP battery between 2003-08. The cohort was prospectively followed with annual telephone contacts, including the Telephone Interview for Cognitive Status (TICS), and 3 in depth neuropsychological evaluations at 5 year intervals in the MRI cohort until 2024. The aging cohort is representative of an elderly, urban, diverse community at risk for cognitive decline. A wealth of data was collected during baseline enrollment and at time of MRI and 1st NP visit, including socio-demographics, psychosocial and socioeconomic status (education, occupational attainment, insurance status), medical history, medications, risk factors, family history and other health data, behavioral/environmental factors, subclinical vascular measures, serum biomarkers (infectious burden, neuroimmune markers using a novel multiplex assay, HOMA index for insulin resistance, adiponectin, CRP, homocysteine), carotid imaging, echocardiographic imaging (LV, LA size), ambulatory BP and cardiac rhythm monitoring, brain MRI biomarkers (regional brain volumes, regional white matter lesion burden, hippocampal volumes, cortical thickness, covert infarcts, cerebral microbleeds, perivascular spaces, brain arterial diameters), and genetic markers (GWAS, ApoE4). Fasting blood was collected and stored at baseline and at MRI. Subjects had complete blood count, chemistry profile, total protein, albumin, calcium, markers of mineral metabolism (fibroblast growth factor 23, parathyroid hormone, 1,25OH and 25OH vitamin D, and phosphate), CRP, TNF receptor levels, IL-6, and serologies against some viral and bacterial pathogens. Fasting plasma levels were assayed for total and HDL cholesterol, lipoprotein (a), HDL particle size, triglycerides, lipoprotein-associated phospholipase A2, homocysteine, serum insulin levels, and adiponectin. Buffy coats and DNA were stored on 2433 subjects and ApoE4

© 2025 Ogungbe O et al. *JAMA Network Open*.

genotype is available on the MRI cohort. Remarkably, only 3 (0.38%) subjects are lost, and 11 (1.4%) have withdrawn from active participation.

Prevent Pulmonary Fibrosis (PrePF): PrePF has been investigating the clinical, physiologic and genetic phenotypes of interstitial lung disease (ILD) by focusing on families with two or more cases of ILD and individuals with sporadic IPF. It has recruited over 1200 families with two or more cases of pulmonary fibrosis. These families with pulmonary fibrosis include 2837 individuals with probable or definite idiopathic interstitial pneumonia (IIP) and 2404 unaffected FDRs. In addition, PrePF recruited over 10,000 individuals with sporadic idiopathic pulmonary fibrosis (IPF).

REasons for Geographic and Racial Differences in Stroke (REGARDS)[15]: the REGARDS cohort is one of the nation's largest, most comprehensive population-based cohorts, its innovative home- and telephone-based data collection is nimble and cost-efficient. REGARDS centrally recruited and initially examined 30,239 non-Hispanic Black and White men and women aged ≥45 years in 2003-7 by telephone and in participant homes across the 48 contiguous US states (62% of US counties). Over 17 years, REGARDS has collected follow-up data by computer-assisted telephone interviews (CATI), participant collaboration in at-home tasks (i.e., actigraphy), and a 2nd in-home visit. REGARDS oversampled Black individuals and residents of the southeastern United States known as the Stroke Belt and 17% reside in rural areas. REGARDS currently follows ~11,000 surviving participants. Comprehensive available data include adjudicated health events, social determinants of health (SDOH), cognition, biomarkers and genomics. Participants currently have mean age 76.9 (range 57-105), with 37% self-identifying as Black, have high cardiovascular risk, and 54% reside in the southeast — all factors associated with COVID-19 risk and adverse outcomes. Participants are geocoded, and linked to external data such as EPA and Medicare. Biorepositories were assembled in 2003-2007 and 2013-2016.

Severe Asthma Research Program (SARP)[16]: SARP has been investigating the clinical, physiologic and molecular phenotypes of asthma since 2000. It is currently following ~400

© 2025 Ogungbe O et al. *JAMA Network Open*.

deeply phenotyped asthma patients (60% severe), most with sputum samples, bronchoscopies, lung CTs, allergy status, spirometry and biobanking.

Subpopulations and Intermediate Outcome Measures in COPD Study (SPIROMICS)[17]: SPIROMICS is a multi-center, observational, longitudinal case-control study designed to guide future development of therapies for COPD by 1) providing robust criteria for sub-classifying COPD participants into groups most likely to benefit from a given therapy during a clinical trial, thereby improving the chances of successful outcome; and 2) identifying biomarkers and phenotypes that can be used as intermediate outcomes to reliably predict clinical benefit during therapeutic trials. The baseline exam included morphometric measures, spirometry, six-minute walk, an inspiratory and expiratory chest CT, and a set of standardized questionnaires. Biospecimens, including plasma, serum, DNA, urine and induced sputum, have been collected and stored. SPIROMICS has recruited 2,983 COPD cases and controls, 40-80 years old with 20+ or <1 pack-years of smoking at 12 US sites in 2010–2015.SPIROMICS has a baseline and 4 follow-up exams, that include spirometry, lung CT scans, sputum induction and, in a subset, bronchoscopies; its current exam is ongoing.

Strong Heart Study (SHS)[18,19]: SHS was designed to respond to the recommendations from the Subcommittee on Cardiovascular and Cerebrovascular Disease of the Secretary of Health and Human Service's Task Force on Black and Minority Health that concluded that information on cardiovascular disease (CVD) in American Indians was inadequate. In its initial stages, the SHS included three components. The first was a survey to determine cardiovascular disease mortality rates from 1984 to 1994 among tribal members aged 35-74 years of age residing in the 3 study areas (the community mortality study). The second was the clinical examination of 4,500 eligible tribal members. The third component is the morbidity and mortality (M&M) surveillance of these 4,500 participants. SHS has completed three clinical examinations of the original Cohort in Phase I 1989-1991; Phase II: 1993-1995; 1998-1999, respectively. In Phases III-V, SHS expanded to include genetic epidemiologic studies and family-based genetics studies due to the importance of genetics in the occurrence of CVD. Phase VI was a surveillance of the original SHS cohort and of the SHS family study participants to better understand CVD, cancer, liver disease, and inflammation in American Indians. Phase VII is currently

© 2025 Ogungbe O et al. *JAMA Network Open*.

underway with continued surveillance beginning February 2019 for a seven-year duration. The SHS Phase VII exam serves as a platform for in-depth ancillary studies that are funded outside of the SHS contracts.

Multiple Imputations

Multiple imputation was used to account for missing covariate data and permit the inclusion of all available C4R participants with non-missing outcome and exposure information. This approach was particularly important as missing data patterns were not random but primarily structural, resulting from differences in data collection across participating cohorts. Specifically, marital status data were not collected in SARP, income data were not available in NOMAS and PrePF, and insurance status was not collected in PrePF, SARP, and SPIROMICS. The multiple imputation procedure was performed using the fully conditional specification method, also known as chained equations, which imputes missing values for each variable iteratively based on a specified set of predictor variables. The imputation model included all variables used in the analysis models, as well as auxiliary variables that were predictive of missingness or the outcome variable. We assumed that data was missing at random. The imputation was conducted using the "mice" package in the R statistical software.[20] Results across 10 imputed datasets were combined by Rubin's Rule.

As a sensitivity analysis, findings from the multiple imputed datasets were compared to a complete case analysis without imputation to assess the impact of missing data on the results (**Supplemental Figure 8**). The consistency between complete case and imputation-based analyses provides reassurance regarding the validity of our findings.

Supplemental Figure 1. CONSORT Diagram of Participants Included in the Analyses

© 2025 Ogungbe O et al. *JAMA Network Open*.

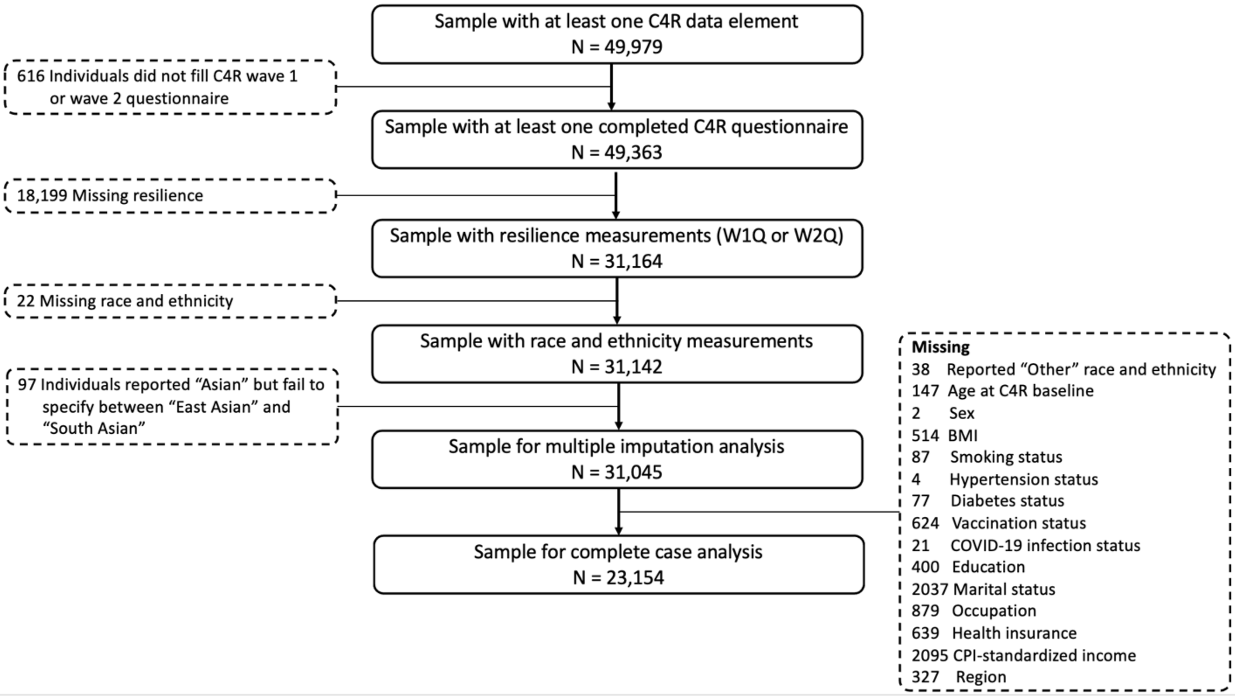

© 2025 Ogungbe O et al. *JAMA Network Open*.

Supplemental Table 1. Association Between Finer Stratification of Age Group and Self-reported Resilience [a]

Variable	Model 1 [b] N = 23,154		Model 2 [b] N = 23,154	
	PR (95% CI)	p-value	PR (95% CI)	p-value
**Age Group**				
Age <45	Reference [b]	NA	1.01 (0.97, 1.06)	0.57
45-54			0.97 (0.93, 1.00)	0.04
55-64			0.98 (0.95, 1.00)	0.04
65-74	1.02 (1.00-1.05)	0.04	Reference [c]	NA
75-84	1.05 (1.02-1.07)	<.001	1.02 (1.00, 1.04)	0.06
Age ≥85	1.02 (0.98-1.05)	0.31	0.99 (0.96, 1.02)	0.67

Statistically significant at p<0.05
[a] Only reporting age associations. Other covariates have identical associations with self-reported resilience.
[b] Model 1 and 2 adjusted for the same set of covariates, including race and ethnicity, age, sex, BMI, smoking, diabetes, hypertension, vaccine status, infection status, education, marital status, occupation, insurance, income and region. However, Model 1 adjusted age as 4 categories (Age<65, 65-74, 75-84, >=85), and Model 2 adjusted age as 6 categories (Age<45, 45-54, 55-64, 65-74, 75-84, >=85). Reference group in Model 1 is Age <65. Reference group in Model 2 is 65<=Age<75.

Supplemental Figure 2. Forest plot of associations of hypertension with self-reported resilience by race and ethnicity

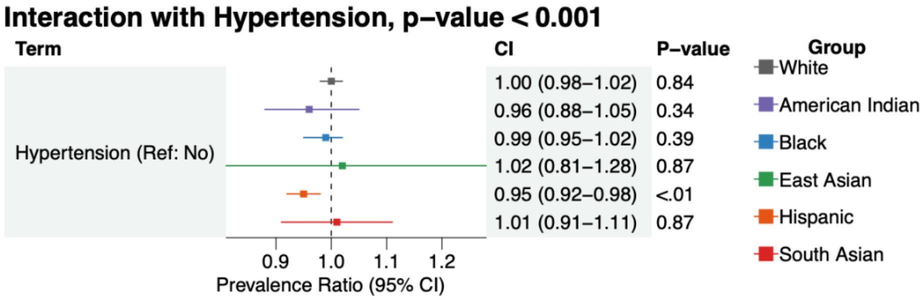

© 2025 Ogungbe O et al. *JAMA Network Open*.

Supplemental Figure 3. Forest plot of associations of region with self-reported resilience by race and ethnicity

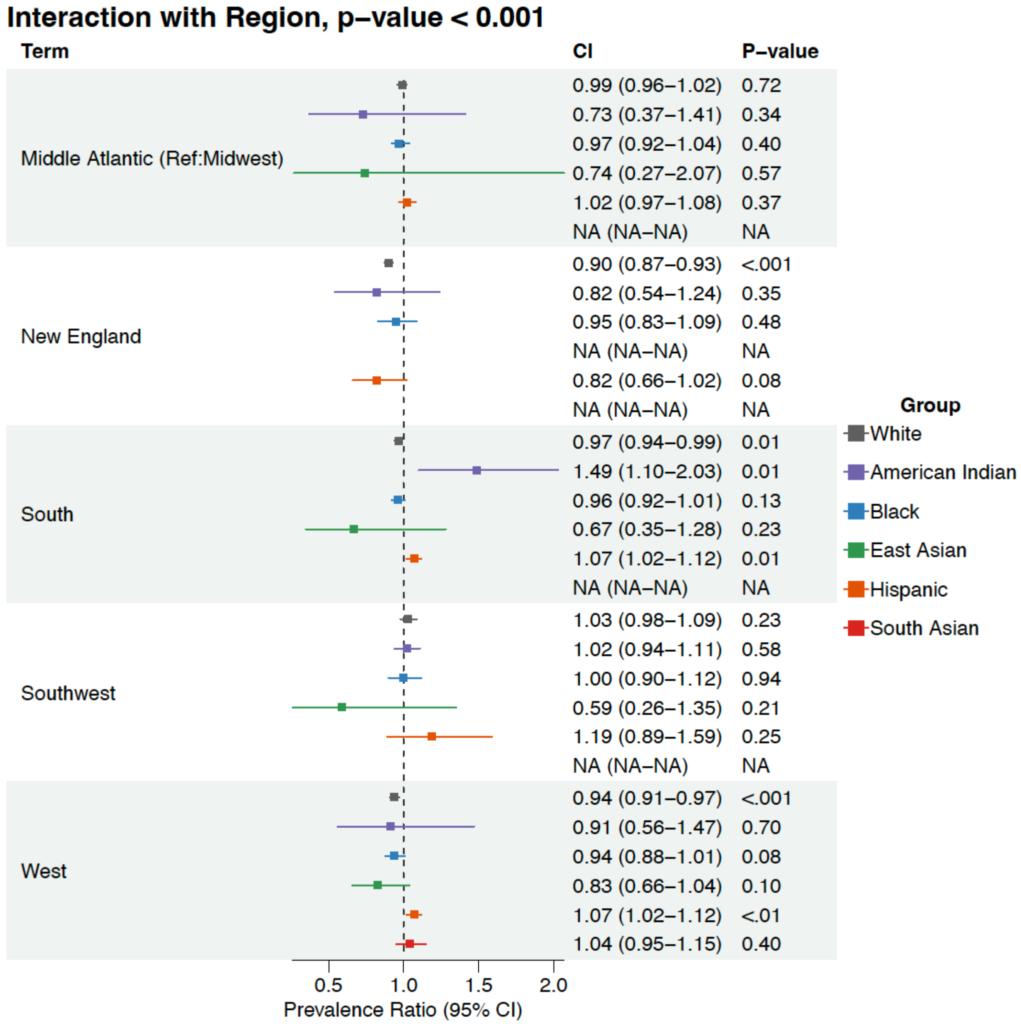

© 2025 Ogungbe O et al. *JAMA Network Open*.

Supplemental Figure 4. Forest plot of associations of insurance with self-reported resilience by race and ethnicity

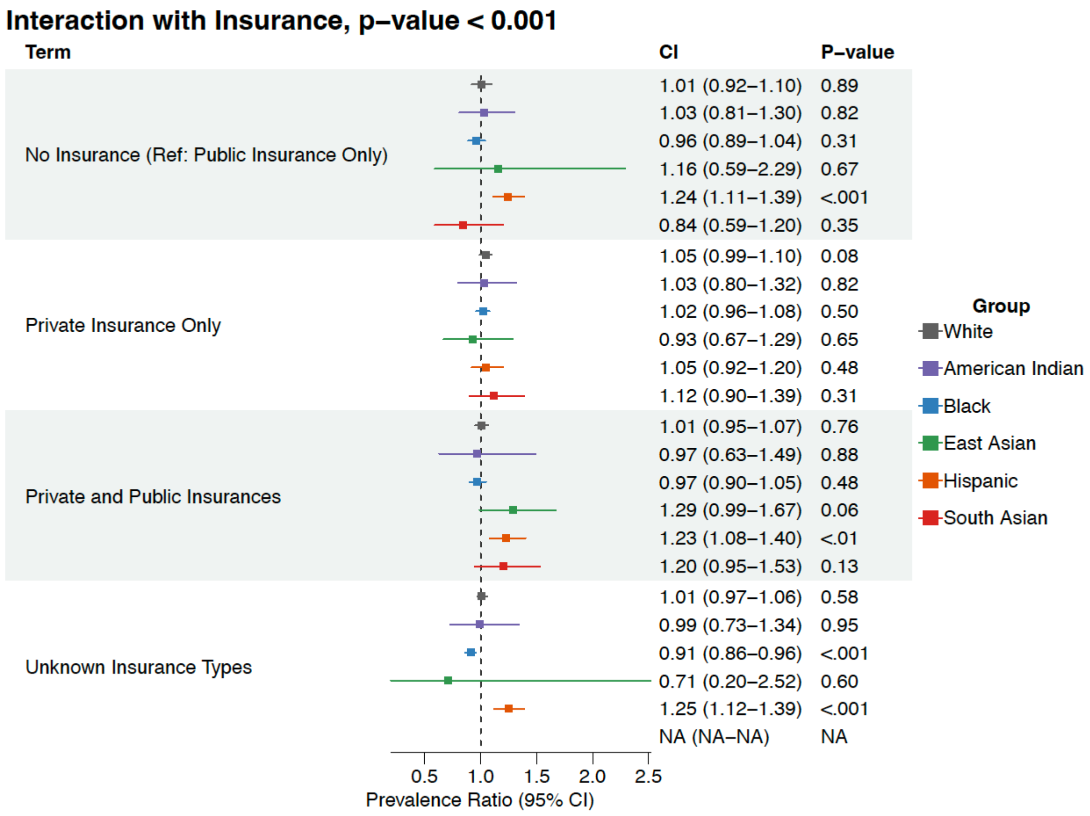

© 2025 Ogungbe O et al. *JAMA Network Open*.

Supplemental Figure 5. Forest plot of associations of BMI categories with self-reported resilience by race and ethnicity

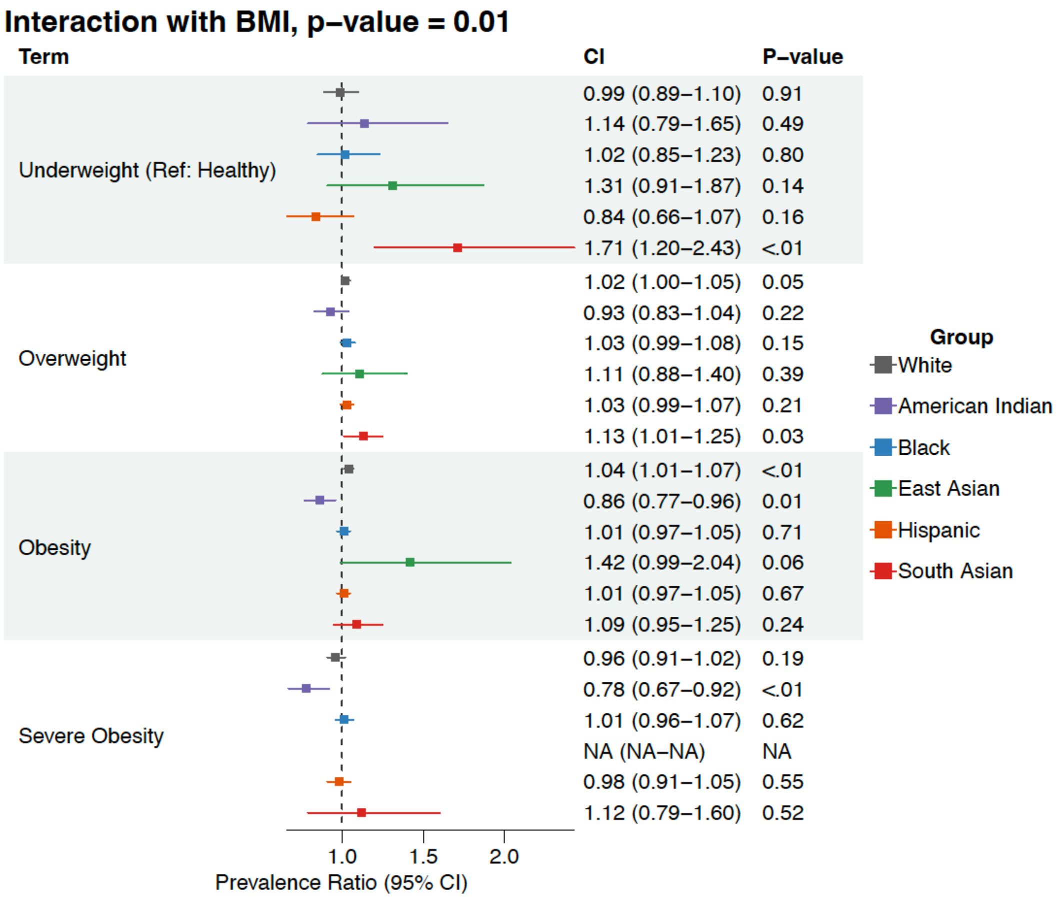

© 2025 Ogungbe O et al. *JAMA Network Open*.

Supplemental Figure 6. Forest plot of associations of diabetes with self-reported resilience by race and ethnicity

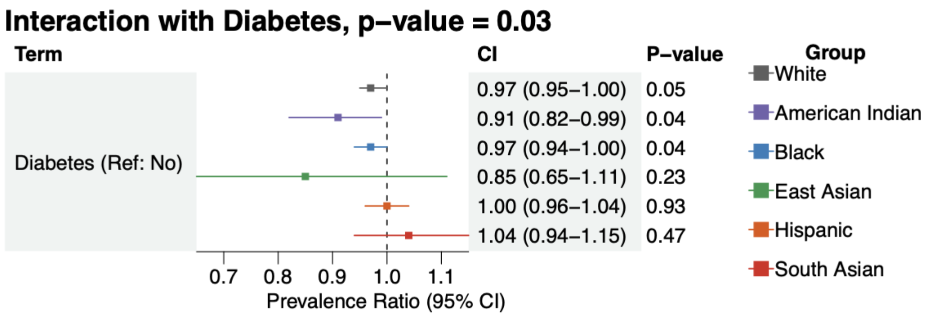

Supplemental Figure 7. Forest plot of associations of marital status with self-reported resilience by race and ethnicity

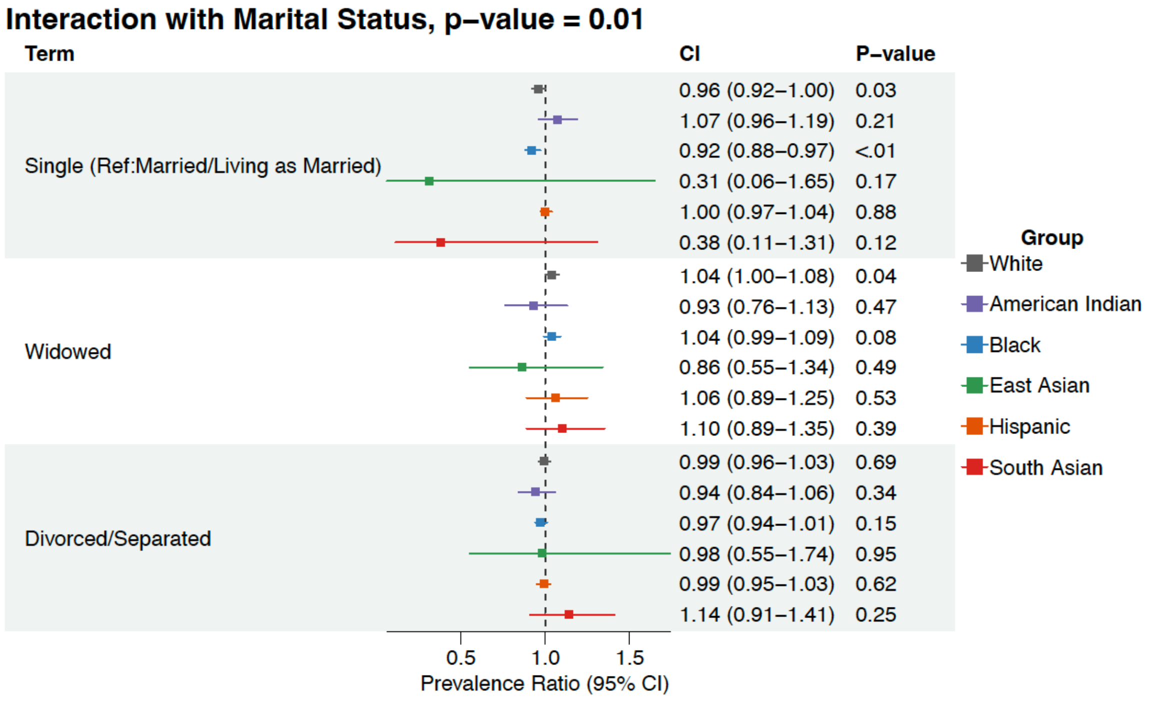

© 2025 Ogungbe O et al. *JAMA Network Open*.

Supplemental Table 2. Participant Characteristics by Self-reported Resilience Status Using Complete Case Data

Sample Characteristics [a]	Response to "I tend to bounce back quickly after hard times"		Total
	Agree or Strongly Agree, "Resilient" (%)[b]	Neutral, Disagree, or Strongly Disagree, "Not Resilient"(%)[b]	
**Classified Events, n**	17397	5757	23154
**Race and Ethnicity [c]**			
White	8470 (48.7)	2929 (50.9)	11399 (49.2)
American Indian	174 (1.0)	95 (1.7)	269 (1.2)
Black	3941 (22.7)	1223 (21.2)	5164 (22.3)
East Asian	158 (0.9)	127 (2.2)	285 (1.2)
Hispanic	4246 (24.4)	1252 (21.7)	5498 (23.7)
South Asian	408 (2.3)	131 (2.3)	539 (2.3)
**Age Group**			
Age<65	6005 (34.5)	1951 (33.9)	7956 (34.4)
65-74	4452 (25.6)	1515 (26.3)	5967 (25.8)
75-84	5018 (28.8)	1604 (27.9)	6622 (28.6)
Age>=	1922 (11.0)	687 (11.9)	2609 (11.3)
**Sex**			
Female	10286 (59.1)	3642 (63.3)	13928 (60.2)
Male	7111 (40.9)	2115 (36.7)	9226 (39.8)
**BMI [d]**			
Underweight	129 (0.7)	48 (0.8)	177 (0.8)
Healthy	3839 (22.1)	1417 (24.6)	5256 (22.7)
Overweight	6654 (38.2)	2050 (35.6)	8704 (37.6)
Obesity	5785 (33.3)	1869 (32.5)	7654 (33.1)
Severe Obesity	990 (5.7)	373 (6.5)	1363 (5.9)
**Smoking Status**			
Never	9330 (53.6)	3069 (53.3)	12399 (53.6)
Former	6433 (37.0)	2051 (35.6)	8484 (36.6)
Current	1634 (9.4)	637 (11.1)	2271 (9.8)
**Diabetes**			
Yes	3865 (22.2)	1414 (24.6)	5279 (22.8)
**Hypertension**			
Yes	10904 (62.7)	3658 (63.5)	14562 (62.9)

© 2025 Ogungbe O et al. *JAMA Network Open*.

**Vaccine Status at Completion of C4R Survey**			
Vaccinated	14882 (85.5)	4941 (85.8)	19823 (85.6)
**COVID-19 Infection at Completion of C4R Survey**			
Infected	2645 (15.2)	829 (14.4)	3474 (15.0)
**Education**			
< High School	2051 (11.8)	793 (13.8)	2844 (12.3)
High School	3656 (21.0)	1291 (22.4)	4947 (21.4)
Some College	3318 (19.1)	1165 (20.2)	4483 (19.4)
College +	8372 (48.1)	2508 (43.6)	10880 (47.0)
**Marital Status**			
Single	1796 (10.3)	646 (11.2)	2442 (10.5)
Married/Living as Married	11439 (65.8)	3620 (62.9)	15059 (65.0)
Widowed	1359 (7.8)	483 (8.4)	1842 (8.0)
Divorced/Separated	2803 (16.1)	1008 (17.5)	3811 (16.5)
**Occupation**			
Employed	9623 (55.3)	2834 (49.2)	12457 (53.8)
**Health Insurance**			
No Insurance	1515 (8.7)	463 (8.0)	1978 (8.5)
Private Insurance Only	4679 (26.9)	1317 (22.9)	5996 (25.9)
Public Insurance Only	746 (4.3)	306 (5.3)	1052 (4.5)
Private and Public Insurances	1167 (6.7)	396 (6.9)	1563 (6.8)
Unknown Type of Insurance [e]	9290 (53.4)	3275 (56.9)	12565 (54.3)
**Household Income** [f]			
<$50,000	8691 (50.0)	3177 (55.2)	11868 (51.3)
$50,000 – 100,000	4306 (24.8)	1362 (23.7)	5668 (24.5)
>$100,000	4400 (25.3)	1218 (21.2)	5618 (24.3)
**Region**			
Middle Atlantic	2471 (14.2)	729 (12.7)	3200 (13.8)
Midwest	3492 (20.1)	1144 (19.9)	4636 (20.0)
New England	1049 (6.0)	454 (7.9)	1503 (6.5)
South	7169 (41.2)	2293 (39.8)	9462 (40.9)
Southwest	385 (2.2)	128 (2.2)	513 (2.2)
West	2831 (16.3)	1009 (17.5)	3840 (16.6)
**Study**			

© 2025 Ogungbe O et al. *JAMA Network Open*.

ARIC	2946 (16.9)	757 (13.1)	3703 (16.0)
CARDIA	1309 (7.5)	428 (7.4)	1737 (7.5)
COPDGene	603 (3.5)	150 (2.6)	753 (3.3)
FHS	1046 (6.0)	436 (7.6)	1482 (6.4)
HCHS	3909 (22.5)	1135 (19.7)	5044 (21.8)
JHS	953 (5.5)	241 (4.2)	1194 (5.2)
MASALA	408 (2.3)	131 (2.3)	539 (2.3)
MESA	1368 (7.9)	494 (8.6)	1862 (8.0)
NOMAS	0 (0.0)	0 (0.0)	0 (0.0)
PrePF	0 (0.0)	0 (0.0)	0 (0.0)
REGARDS	4691 (27.0)	1897 (33.0)	6588 (28.5)
SARP	0 (0.0)	0 (0.0)	0 (0.0)
SHS	164 (0.9)	88 (1.5)	252 (1.1)
SPIROMICS	0 (0.0)	0 (0.0)	0 (0.0)

[a] All counts and column percentages come from complete case dataset.
[b] W1Q resilience is favored when participants have both W1Q and W2Q measurements. We adopted the first record of resilience. Additionally, we combined the original "Strongly Disagree," "Disagree" and "Neutral" groups into the new "Disagree" group, the original "Strongly Agree" and "Agree" groups into the new "Agree" group.
[c] Asian participants in cohorts other than MASALA or MESA were dropped due to unclear race and ethnicity measurements and small sample sizes.
[d] BMI categories are underweight (<18.5), healthy weight (18.5-24.9), overweight (25.0-29.9), obesity (30.0-39.9), and severe obesity (>=40.0).
[e] Participants have insurance, but the exact insurance type is unknown
[f] Income is standardized into 2020 dollars using customer price index (CPI, CPI_{2020} = 258.811.)

© 2025 Ogungbe O et al. *JAMA Network Open*.

Supplemental Table 3. Multivariable-adjusted Associations with Self-reported Resilience Using Complete Case Data

Variable (Reference)	Model 1<br>N = 23,154		Model 2<br>N = 23,154	
	PR (95% CI)	p-value	PR (95% CI)	p-value
**Race and Ethnicity (White)**				
American Indian	0.87 (0.79-0.95)	<.01	0.86 (0.78-0.94)	<.01
Black	1.03 (1.01-1.05)	<.001	1.04 (1.02-1.07)	<.001
East Asian	0.75 (0.67-0.83)	<.001	0.76 (0.69-0.85)	<.001
Hispanic	1.05 (1.03-1.07)	<.001	1.13 (1.10-1.16)	<.001
South Asian	1.03 (0.98-1.08)	0.30	0.96 (0.90-1.01)	0.10
**Age Group (Age<65)**				
65-74	1.00 (0.98-1.02)	0.82	1.02 (1.00-1.05)	0.04
75-84	1.03 (1.01-1.05)	0.01	1.05 (1.02-1.07)	<.001
Age>=85	1.00 (0.97-1.03)	0.94	1.02 (0.98-1.05)	0.31
**Sex (Male)**				
Female	0.95 (0.94-0.97)	<.001	0.97 (0.96-0.99)	<.001
**BMI (Healthy)**				
Underweight			1.02 (0.93-1.12)	0.70
Overweight			1.03 (1.01-1.06)	<.01
Obesity			1.03 (1.01-1.05)	0.01
Severe Obesity			1.00 (0.96-1.04)	0.92
**Smoking Status (Never)**				
Former			1.01 (0.99-1.03)	0.22
Current			0.97 (0.94-1.00)	0.02
**Diabetes Status (No)**				
Yes			0.97 (0.96-0.99)	<.01
**Hypertension Status (No)**				
Yes			0.98 (0.97-1.00)	0.09
**Vaccine Status at Completion of C4R Survey (Not Vaccinated)**				
Vaccinated			0.98 (0.96-1.00)	0.04
**COVID-19 Infection at Completion of C4R Survey (Not Infected)**				
Infected			1.00 (0.98-1.03)	0.75

© 2025 Ogungbe O et al. *JAMA Network Open*.

**Education (<High School)**				
High School			1.04 (1.01-1.07)	0.01
Some College			1.06 (1.02-1.09)	<.001
College +			1.08 (1.05-1.11)	<.001
**Marital Status (Married/Living as Married)**				
Single			0.97 (0.94-0.99)	0.02
Widowed			1.03 (1.00-1.06)	0.05
Divorced/Separated			0.98 (0.96-1.00)	0.11
**Occupation (Employed)**				
Other than Employed			0.95 (0.93-0.97)	<.001
**Health Insurance (Public Insurance Only)**				
No Insurance			1.01 (0.96-1.06)	0.74
Private Insurance Only			1.04 (1.00-1.09)	0.06
Private and Public Insurances			1.02 (0.97-1.07)	0.50
Unknown Type of Insurance			0.98 (0.94-1.02)	0.26
**Income (<$50,000)**				
$50,000-100,000			1.03 (1.01-1.05)	<.01
>$100,000			1.07 (1.05-1.10)	<.001
**Region (Midwest)**				
Middle Atlantic			1.02 (1.00-1.05)	0.10
New England			0.89 (0.85-0.93)	<.001
South			1.00 (0.97-1.02)	0.65
Southwest			1.04 (0.98-1.09)	0.19
West			0.98 (0.95-1.00)	0.07
Statistically significant at p<0.05				

© 2025 Ogungbe O et al. *JAMA Network Open*.

Supplemental Figure 8. Forest plot showing correlates of self-reported resilience, comparing results from the multiple imputed analyses to the complete case analyses

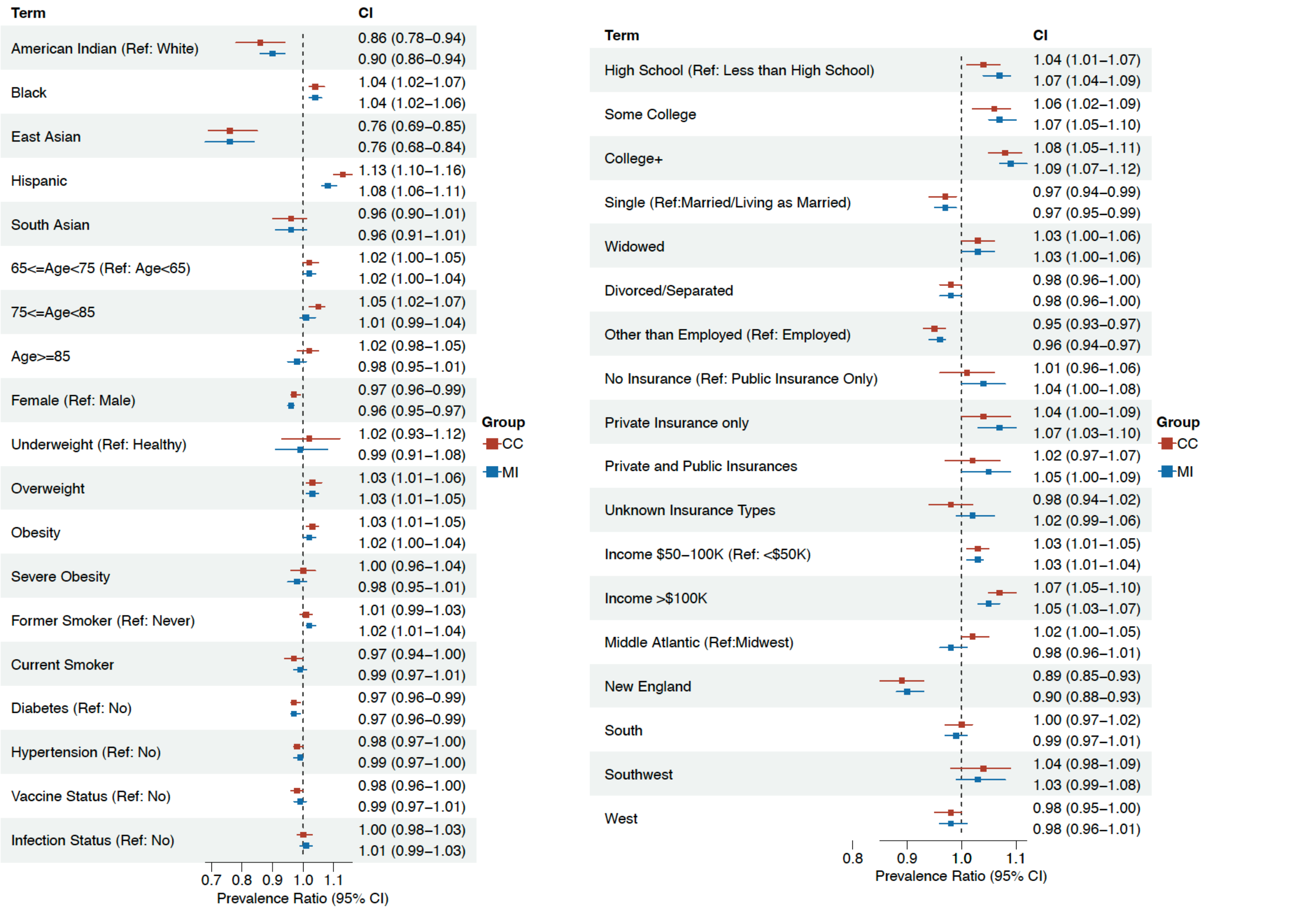

© 2025 Ogungbe O et al. *JAMA Network Open*.

Supplemental Table 4. Multivariable-adjusted associations with self-reported resilience, adjusting for the time between covariate measurement and resilience assessment

Variable (Reference)	Model 1 N = 23,154		Model 2 [a] N = 19,845	
	PR (95% CI)	p-value	PR (95% CI)	p-value
**Time Variable for Marital Status (tt_marital)**	Not Adjusted		1.00 (0.99-1.00)	0.65
**Time Variable for Occupation (tt_job)**	Not Adjusted		1.00 (0.99-1.00)	0.05
**Time Variable for Insurance (tt_insurance)**	Not Adjusted		1.00 (1.00-1.01)	0.23
**Time Variable for Income (tt_income)**	Not Adjusted		1.00 (1.00-1.01)	0.02
**Race and Ethnicity (White)**				
American Indian	0.86 (0.78-0.94)	<.01	0.73 (0.62-0.85)	<.001
Black	1.04 (1.02-1.07)	<.001	1.04 (1.02-1.07)	<.001
East Asian	0.76 (0.69-0.85)	<.001	0.76 (0.68-0.85)	<.001
Hispanic	1.13 (1.10-1.16)	<.001	1.12 (1.08-1.16)	<.001
South Asian	0.96 (0.90-1.01)	0.10	1.01 (0.94-1.07)	0.86
**Age Group (Age <65)**				
65-74	1.02 (1.00-1.05)	0.04	1.03 (0.99-1.07)	0.13
75-84	1.05 (1.02-1.07)	<.001	1.03 (1.00-1.06)	0.05
Age ≥85	1.02 (0.98-1.05)	0.31	0.99 (0.95-1.04)	0.78
**Sex (Male)**				
Female	0.97 (0.96-0.99)	<.001	0.97 (0.96-0.99)	<.01
**BMI (Healthy)**				
Underweight	1.02 (0.93-1.12)	0.70	1.01 (0.92-1.12)	0.80
Overweight	1.03 (1.01-1.06)	<.01	1.03 (1.01-1.06)	<.01
Obesity	1.03 (1.01-1.05)	0.01	1.03 (1.01-1.05)	0.02
Severe Obesity	1.00 (0.96-1.04)	0.92	1.00 (0.96-1.05)	0.89
**Smoking Status (Never)**				
Former	1.01 (0.99-1.03)	0.22	1.01 (0.99-1.03)	0.33
Current	0.97 (0.94-1.00)	0.02	0.97 (0.94-1.00)	0.04
**Diabetes Status (No)**				
Yes	0.97 (0.96-0.99)	<.01	0.98 (0.96-1.00)	0.07
**Hypertension Status (No)**				
Yes	0.98 (0.97-1.00)	0.09	0.98 (0.96-1.00)	0.02
**Vaccine Status at Completion of C4R Survey (Not Vaccinated)**				
Vaccinated	0.98 (0.96-1.00)	0.04	0.98 (0.96-1.01)	0.19
**COVID-19 Infection at Completion of C4R Survey (Not Infected)**				
Infected	1.00 (0.98-1.03)	0.75	1.00 (0.98-1.03)	0.81
**Education (<High School)**				
High School	1.04 (1.01-1.07)	0.01	1.04 (1.01-1.07)	0.01
Some College	1.06 (1.02-1.09)	<.001	1.05 (1.02-1.09)	<.01
College +	1.08 (1.05-1.11)	<.001	1.08 (1.04-1.11)	<.001

© 2025 Ogungbe O et al. *JAMA Network Open*.

**Marital Status (Married/Living as Married)**				
Single	0.97 (0.94-0.99)	0.02	0.97 (0.90-1.04)	0.42
Widowed	1.03 (1.00-1.06)	0.05	1.03 (0.98-1.09)	0.26
Divorced/Separated	0.98 (0.96-1.00)	0.11	0.96 (0.91-1.00)	0.06
**Occupation (Employed)**				
Other than Employed	0.95 (0.93-0.97)	<.001	0.94 (0.91-0.97)	<.001
**Health Insurance (Public Insurance Only)**				
No Insurance	1.01 (0.96-1.06)	0.74	0.98 (0.91-1.06)	0.62
Private Insurance Only	1.04 (1.00-1.09)	0.06	0.96 (0.89-1.03)	0.26
Private and Public Insurances	1.02 (0.97-1.07)	0.50	1.00 (0.93-1.08)	0.95
Unknown Type of Insurance	0.98 (0.94-1.02)	0.26	0.98 (0.92-1.05)	0.63
**Income (<$50,000)**				
$50,000-100,000	1.03 (1.01-1.05)	<.01	1.05 (1.00-1.09)	0.03
>$100,000	1.07 (1.05-1.10)	<.001	1.08 (1.04-1.13)	<.001
**Region (Midwest)**				
Middle Atlantic	1.02 (1.00-1.05)	0.10	1.03 (1.01-1.06)	0.02
New England	0.89 (0.85-0.93)	<.001	0.94 (0.90-0.99)	0.02
South	1.00 (0.97-1.02)	0.65	1.01 (0.99-1.04)	0.25
Southwest	1.04 (0.98-1.09)	0.19	1.09 (1.03-1.15)	<.01
West	0.98 (0.95-1.00)	0.07	0.99 (0.97-1.02)	0.66
**Interaction Terms**				
tt_marital × Single	Not Adjusted		1.00 (0.99-1.01)	0.83
tt_marital × Widowed	Not Adjusted		1.00 (0.99-1.00)	0.58
tt_marital × Divorced/Separated	Not Adjusted		1.00 (0.99-1.00)	0.18
tt_job × Other than Employed	Not Adjusted		1.00 (1.00-1.00)	0.10
tt_insurance × No Insurance	Not Adjusted		0.99 (0.99-1.00)	0.10
tt_insurance × Private Only	Not Adjusted		0.99 (0.99-1.00)	0.06
tt_insurance × Private and Public	Not Adjusted		1.00 (0.99-1.00)	0.34
tt_insurance × Unknown Types	Not Adjusted		1.00 (0.99-1.00)	0.64
tt_income × $50-100K	Not Adjusted		1.00 (1.00-1.01)	0.38
tt_income × 100K+	Not Adjusted		1.00 (1.00-1.00)	0.84

Statistically significant at $p<0.05$
[a] Model 2 (N =19,845) used a subset of the Model 1 (N = 23,154) sample due to missing time to measurement data. Model 2 additionally adjusted for four time-to-measurement variables and their interactions with corresponding SDOH variables.

© 2025 Ogungbe O et al. *JAMA Network Open*.

eReferences

1. The Atherosclerosis Risk in Communities (ARIC) Study: design and objectives. The ARIC investigators. *Am J Epidemiol*. 1989;129(4):687-702.

2. Friedman GD, Cutter GR, Donahue RP, et al. CARDIA: study design, recruitment, and some characteristics of the examined subjects. *J Clin Epidemiol*. 1988;41(11):1105-1116. doi:10.1016/0895-4356(88)90080-7

3. Regan EA, Hokanson JE, Murphy JR, et al. Genetic epidemiology of COPD (COPDGene) study design. *COPD*. 2010;7(1):32-43. doi:10.3109/15412550903499522

4. Tsao CW, Vasan RS. Cohort Profile: The Framingham Heart Study (FHS): overview of milestones in cardiovascular epidemiology. *Int J Epidemiol*. 2015;44(6):1800-1813. doi:10.1093/ije/dyv337

5. Lavange LM, Kalsbeek WD, Sorlie PD, et al. Sample design and cohort selection in the Hispanic Community Health Study/Study of Latinos. *Ann Epidemiol*. 2010;20(8):642-649. doi:10.1016/j.annepidem.2010.05.006

6. Sorlie PD, Avilés-Santa LM, Wassertheil-Smoller S, et al. Design and implementation of the Hispanic Community Health Study/Study of Latinos. *Ann Epidemiol*. 2010;20(8):629-641. doi:10.1016/j.annepidem.2010.03.015

7. Daviglus ML, Talavera GA, Avilés-Santa ML, et al. Prevalence of Major Cardiovascular Risk Factors and Cardiovascular Diseases Among Hispanic/Latino Individuals of Diverse Backgrounds in the United States. *JAMA*. 2012;308(17):1775. doi:10.1001/jama.2012.14517

8. Carpenter MA, Crow R, Steffes M, et al. Laboratory, reading center, and coordinating center data management methods in the Jackson Heart Study. *Am J Med Sci*. 2004;328(3):131-144. doi:10.1097/00000441-200409000-00001

9. Taylor HA, Wilson JG, Jones DW, et al. Toward resolution of cardiovascular health disparities in African Americans: design and methods of the Jackson Heart Study. *Ethn Dis*. 2005;15(4 Suppl 6):S6-4-17.

10. Keku E, Rosamond W, Taylor HA, et al. Cardiovascular disease event classification in the Jackson Heart Study: methods and procedures. *Ethn Dis*. 2005;15(4 Suppl 6):S6-62-70.

11. Kanaya AM, Kandula N, Herrington D, et al. Mediators of Atherosclerosis in South Asians Living in America (MASALA) study: objectives, methods, and cohort description. *Clin Cardiol*. 2013;36(12):713-720. doi:10.1002/clc.22219

12. Kanaya AM, Chang A, Schembri M, et al. Recruitment and retention of US South Asians for an epidemiologic cohort: Experience from the MASALA study. *J Clin Transl Sci*. 2019;3(2-3):97-104. doi:10.1017/cts.2019.371

13. Bild DE. Multi-Ethnic Study of Atherosclerosis: Objectives and Design. *Am J Epidemiol*. 2002;156(9):871-881. doi:10.1093/aje/kwf113

© 2025 Ogungbe O et al. *JAMA Network Open*.

14. Sacco RL, Boden-Albala B, Gan R, et al. Stroke incidence among white, black, and Hispanic residents of an urban community: the Northern Manhattan Stroke Study. *Am J Epidemiol*. 1998;147(3):259-268. doi:10.1093/oxfordjournals.aje.a009445

15. Howard VJ, Cushman M, Pulley L, et al. The reasons for geographic and racial differences in stroke study: objectives and design. *Neuroepidemiology*. 2005;25(3):135-143. doi:10.1159/000086678

16. Teague WG, Phillips BR, Fahy JV, et al. Baseline Features of the Severe Asthma Research Program (SARP III) Cohort: Differences with Age. *J Allergy Clin Immunol Pract*. 2018;6(2):545-554.e4. doi:10.1016/j.jaip.2017.05.032

17. Couper D, LaVange LM, Han M, et al. Design of the Subpopulations and Intermediate Outcomes in COPD Study (SPIROMICS). *Thorax*. 2014;69(5):491-494. doi:10.1136/thoraxjnl-2013-203897

18. Lee ET, Welty TK, Fabsitz R, et al. The Strong Heart Study. A study of cardiovascular disease in American Indians: design and methods. *Am J Epidemiol*. 1990;132(6):1141-1155. doi:10.1093/oxfordjournals.aje.a115757

19. North KE, Howard BV, Welty TK, et al. Genetic and environmental contributions to cardiovascular disease risk in American Indians: the strong heart family study. *Am J Epidemiol*. 2003;157(4):303-314. doi:10.1093/aje/kwf208

20. Buuren SV, Groothuis-Oudshoorn K. MICE: Multivariate Imputation by Chained Equations in R. *J Stat Softw*. 2011;45(3). doi:10.18637/jss.v045.i03

© 2025 Ogungbe O et al. *JAMA Network Open*.
