# Supplementary material for: Self-Reported Resilience During the COVID-19 Pandemic
Source: JAMA Netw Open. 2025 Jul 16;8(7):e2520360. doi: 10.1001/jamanetworkopen.2025.20360 (PMC12268485; doi:10.1001/jamanetworkopen.2025.20360)
Supplement: Supplement 2. — Data Sharing Statement [file jamanetwopen-e2520360-s002.pdf]

Data Sharing Statement

Ogungbe. Self-Reported Resilience During the COVID-19 Pandemic. *JAMA Netw Open*. Published July 16, 2025. doi:10.1001/jamanetworkopen.2025.20360

Data
Data available: No
